# Invasion genetics of the silver carp *Hypophthalmichthys molitrix* across North America: Differentiation of fronts, introgression, and eDNA metabarcode detection

**DOI:** 10.1371/journal.pone.0203012

**Published:** 2019-03-27

**Authors:** Carol A. Stepien, Matthew R. Snyder, Anna E. Elz

**Affiliations:** NOAA Pacific Marine Environmental Laboratory, Genetics and Genomics Group (G3), Seattle, WA, United States of America; National Cheng Kung University, TAIWAN

## Abstract

In the 1970s, the introduced silver carp *Hypophthalmichthys molitrix* (which is indigenous to eastern Asia) escaped from southern U.S. aquaculture to spread throughout the Mississippi River basin, and since has steadily moved northward. This large, prolific filter-feeder reduces food availability for other fishes. It now has reached the threshold of the Laurentian Great Lakes, where it likely will significantly impact food chains and fisheries. Our study evaluates population genetic variability and differentiation of the silver carp using 10 nuclear DNA microsatellite loci, and sequences of two mitochondrial genes–cytochrome *b* and cytochrome *c* oxidase subunit 1, along with the nuclear ribosomal protein S7 gene intron 1. We analyze population samples from: two primary Great Lakes’ invasion fronts (at the Illinois River outside of Chicago, IL in Lake Michigan and in the Wabash River, which leads into the Maumee River and western Lake Erie), the original establishment “core” in the Lower Mississippi River, and expansion areas in the Upper Mississippi and Missouri rivers. We analyze and compare our results with bighead and other invasive carps, and cyprinid relatives. Results reveal that the silver carp invasion possesses moderate levels of genetic diversity, with more mtDNA haplotypes and unique microsatellite alleles in the “core” Lower Mississippi River population, which also diverges the most. The two invasion fronts also significantly genetically differ. About 3% of individuals (including all populations except the Illinois River) contain a unique and very divergent mtDNA haplotype, which likely stems from historic introgression in Asia with female largescale silver carp *H*. *harmandi*. The nuclear microsatellites and S7 sequences of the introgressed individuals do not differ from silver carp and are very distant from bighead carp. These sequence variation data are employed to design and evaluate a targeted high-throughput metabarcoding sequence assay that identifies and distinguishes among species of invasive carps (i.e., silver, bighead, grass, black, and common carps, along with goldfish), as well as native cyprinids, using cytochrome *b*. Our assay further differentiates among selected silver carp haplotypes (including between *H*. *molitrix* and *H*. *harmandi*), for use in population genetics and future analyses of spread pathways. We test and evaluate this assay on environmental (e)DNA water samples from 48 bait shops in the Great Lakes’ region (along the Lake Erie, Lake St. Clair, and Wabash River watersheds), using positive and negative controls and custom bioinformatic processing. Test results discern silver carp eDNA in four of the shops–three in Lake Erie and one in the Wabash River watershed–and bighead carp from one of the same Lake Erie venues, suggesting that retailers (who often source from established southerly populations) comprise another introduction vector. Our overall findings thus provide key population genetic and phylogenetic data for understanding and tracing introductions, vectors, and spread pathways for silver carp, their variants, and their relatives.

## Introduction

Discerning the population genetic trajectories of nonindigenous species invasions can enhance our overall understanding of the evolutionary adaptations and changing ecological community dynamics governing today’s ecosystems [1–3]. Establishments by invasive species comprise accidental experiments to ground-truth evolutionary and ecological theory with reality [4–6]. The genetic variation of invasive populations often influences their relative success and persistence in new habitats, including colonizing, reproducing, spreading, and overcoming biotic resistance [2, 7, 8].

Traditional invasion theory predicted that most introduced populations would be characterized by low genetic diversity due to founder effect, which would limit adaptive potential [9–11]. More recently, some invasions founded by large numbers of introduced propagules, multiple events, and multiple sources have been found to possess as high or even higher levels of population genetic variability than those in native regions, due to admixture [12–14]. For example, ballast water introductions from Eurasian sources into the North American Laurentian Great Lakes of the zebra mussel *Dreissena polymorpha* (Pallas, 1771) [15, 16], the quagga mussel *D*. *rostriformis* (Deshayes, 1838) [15, 16], and the round goby *Neogobius melanostomus* (Pallas, 1814) [5, 17–19] all possessed relatively high population genetic diversities and significant population divergences across their introduced ranges, on par with those of native populations. These invasions stemmed from large numbers of introduced propagules and multiple introduction events, which have been traced to several founding sources [5, 15–19]. Such genetic variability may enhance adaptive potential of invasions (see [11]).

The “leading edge” hypothesis postulates that expansion populations at an invasion’s front(s) should possess less genetic variability than those at the invasion’s core (i.e., the original successful founding area) [20, 21]. According to that hypothesis, individuals at the front(s) likely would be adapted for dispersal and high reproductive output [22], as they would experience low population density, greater resource availability, and less competition, thereby enhancing their relative reproductive success [23, 24]. This might lead to population genetic differences between the expansion fronts versus longer-established core areas, due to drift and/or selection. However, studies of these long-term trends across the spatial and temporal courses of invasions are relatively rare in the literature [5, 6, 22].

Closely related species, which may interact competitively, sometimes are introduced together or in close succession, as in the mid-1980s cases of zebra and quagga mussels in the Great Lakes [25–27]. The quagga mussel typically overtakes the zebra mussel over time, with populations becoming dominated by the former except in shallow areas [28, 29]. In another Great Lakes’ example, the Eurasian round goby and the tubenose *Proterorhinus semilunaris* (Heckel, 1837) goby co-appeared in the 1990s [30], with the round goby consistently remaining more numerous and widespread [31]. In the 1960s and 1970s, the closely-related east-Asian silver carp *Hypophthalmichthys molitrix* (Valenciennes, 1884) and the bighead carp *H*. *nobilis* (Richardson, 1845) both were introduced to control algae in southern U.S. aquaculture endeavors [32]. Hybridization of the two species is relatively common in invasive areas in Asia, Europe, and the U.S. [33, 34], which might lead to an “invasive swarm” having increased “hybrid vigor” [35, 36].

Invasive species of carps are part of a long history of intentional and accidental introductions that seriously threaten the ecological integrity of the Great Lakes, which is the world’s largest freshwater ecosystem [37] as well as one of the most heavily invaded, with >186 established exotic species [38]. Those that have exerted significant ecological impacts include: the sea lamprey *Petromyzon marinus* Linnaeus, 1758 [39], common carp *Cyprinus carpio* Linnaeus, 1758 [40], zebra and quagga mussels [16], round goby [41], spiny waterflea *Bythotrephes longimanus* Leydig, 1860 [42], and rusty crayfish *Orconectes rusticus* (Girard, 1852) [43]. Ricciardi 2001 [44] postulated that such history of multiple invasions may increase the relative chances of success by new arrivals through “invasional meltdown”, which has been disputed by some ecologists [45, 46]. However, it is possible that such “meltdown” [44] and/or common phylogenetic history and ecological characters [7] may have facilitated the joint establishment successes of many introduced cyprinid fishes–collectively termed “carps”. Invasive carps in North America include the goldfish *Carassius auratus* (Linnaeus, 1758), common carp, grass carp *Ctenopharyngodon idella* (Valenciennes in Cuvier and Valenciennes, 1844), black carp *Mylopharyngodon piceus* (Richardson, 1846), and silver and bighead carps.

### Invasion histories of the silver carp and its relatives

**Fig 1:**
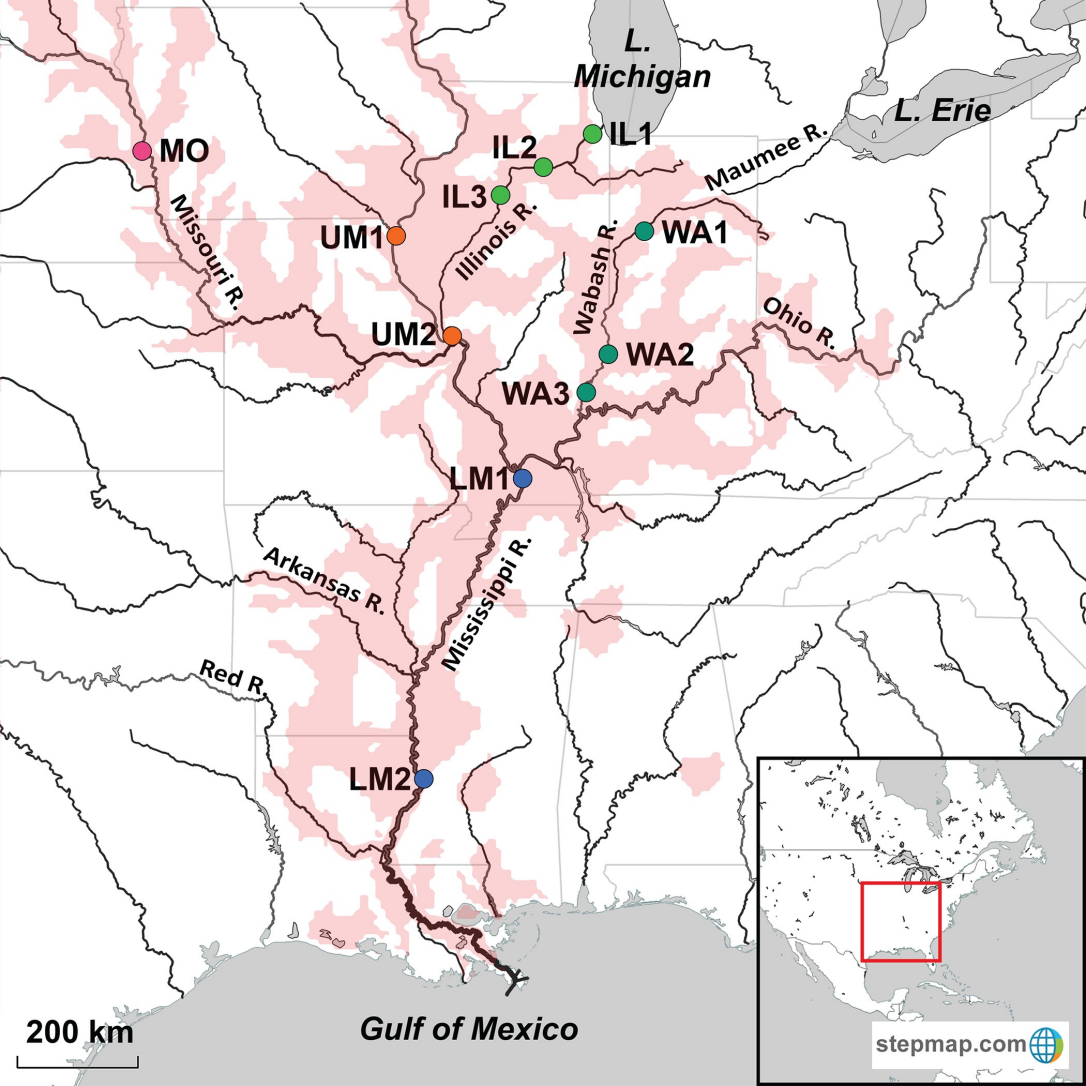
Map of silver carp invasive range (red) and sampling locations (circles, colored by waterway). (IL) Illinois R. IL1: LaGrange, IL (41.757623, -87.849964), IL2: Marseilles, IL (41.322269, -88.707172), IL3: Chillicothe, IL (40.929296, -89.461653). (WA) Wabash R. WA1: Lafayette, IN (40.430177, -86.898111), WA2: Vincennes, IN (38.688718, -87.526298), WA3: New Harmony, IN (38.135032, -87.940739). MO: Missouri R., Blair, NE (41.545091, -96.095555). (UM) Upper Mississippi. UM1: Warsaw, IL. (40.362244, -91.444024), UM2: Grafton, IL (38.966248, -90.430843). (LM) Lower Mississippi. LM1: Laketon, KY (36.868076, -89.124098), LM2: Vicksburg, MS (32.331320, -90.898628). Map created using StepMap.com, with their permission to use.

Many species belonging to the largest freshwater fish family Cyprinidae have been widely introduced in many areas of the world–largely for food and aquaculture–and then escaped to become deleterious invaders, spreading in their new ecosystems, and often competing with native species for food and habitat [47]. In the topical case of the present study, silver and bighead carps both were raised at six state, federal, and private aquaculture facilities in Arkansas during the 1970s, and stocked into municipal sewage lagoons [48]. They then escaped to become established in the Mississippi River basin [32], and since have spread through the upper Mississippi River system [49] (see Fig 1). The two species now are in the Illinois River system outside of Lake Michigan (near Chicago, IL), raising growing concern that they very likely will enter and become established in the Great Lakes [50, 51].

Several other invasive cyprinid fishes (which originally were from Asia, with many having centuries ago been widely introduced in Europe (e.g., [52, 53]), also have long been established in North America [47]. Among these, the goldfish was the first to be intentionally introduced in the late 1600s by settlers with aspiration that this ornamental species would become part of the native fauna [54]. In the early 1800s, the common carp was brought by European settlers into the Great Lakes and other areas for use as a popular food fish [55]. The grass carp was introduced to control vegetation in aquaculture in 1963, and subsequently escaped, similar to the silver and bighead carps [56]. Grass carp remains in use today for controlling algae in ponds, although in the last two decades suppliers have rendered (most) individuals triploid, and (when triploid) unable to reproduce [57, 58]. However, diploid reproductive grass carp have been discerned in Lake Erie [59]. The black carp is believed to have been introduced in the 1970s as a “contaminant” in grass carp stocks in Arkansas [60], and now is established in U.S. states along the Mississippi River south of Illinois [61].

### Invasion ecology of the silver carp and its relatives

Silver and bighead carps are prolific filter-feeders that significantly alter both the numbers of plankters and their community compositions, reducing food for sport and commercial fishes [62–65]. Silver and bighead carps often swim just below the surface, and can travel in large schools (either as single species or together) [66]. In their native ranges, they mature at age 4–8 years, but as early as 2 years old in North America, with each female laying up to 5 million eggs annually [67]. Their reproductive season is longer in the Mississippi River system than in their native habitats [68]. The eggs are buoyant, hatch in one day, and the larvae readily disperse with currents [66]. Adults live to 20 years, reaching >1 m and 27.3 kg [66]. Modeling studies suggest that even a few reproductive individuals could establish successful populations in the Great Lakes [69].

The silver carp’s current North American geographic range is depicted in Fig 1 [70]. Throughout the Mississippi River drainage, its populations extend upstream and downstream of 23 locks and dams (three in the Arkansas River, seven in the Illinois River, eight in the Mississippi River, and five in the Ohio River). At present, there are two potential man-made impediments to silver and bighead carps reaching the Great Lakes basin, of which the first is an electric barrier in the Chicago Area Waterway System that separates the Illinois River from Lake Michigan [71]. This “barrier” frequently is breached by small fishes and larger ones traveling in the wakes of large boats [71]. In 2016, a 2.7 km long, 2.3 m-tall earthen berm was completed in Eagle Marsh at Fort Wayne, IN, between the Wabash and Maumee Rivers (the latter leads into Lake Erie) [72]. This wetland area frequently experienced flooding and connection between the two watersheds, and previously was separated by just a chain link fence, which smaller fishes (and young carps) could readily swim through [72]. The present investigation analyzes populations at these two invasion front areas (i.e., the Illinois and Wabash Rivers), in comparison to genetic variation throughout much of the range. The issue of silver and bighead carps potentially entering and establishing in the Great Lakes is of great concern to managers, the commercial and sport fishing communities, ecologists, and many of the general public [73, 74].

### Research objectives

Little is known about the population genetic and genomic patterns of the silver carp in North America (e.g., [75, 76]). Thus, our objectives are to evaluate genetic diversity and its distribution, and to test for possible population structure. Population samples include the seat of its original establishment in the Mississippi River basin, expansion areas in the Upper Mississippi and the Missouri rivers, and the two most likely invasion fronts leading to the Great Lakes–the Illinois and Wabash rivers. We test invasion genetics theory, including possible founder effect and correspondence to the leading edge hypothesis at the front areas. We further analyze whether population genetic divergence and differentiation occurs across the range, including between the fronts. We evaluate genetic variation using 10 nuclear (n)DNA microsatellite (μsat) loci and sequences from two mitochondrial (mt) genes, which include 992 nucleotides (nts) of cytochrome *b* (cyt*b*) and 549 nts of the cytochrome oxidase subunit I (COI). We compare representative subsets encompassing all mt and μsat variants with nDNA sequence variation from the single copy ribosomal protein S7 gene intron 1. We relate our results to other genetic studies of silver carp, its relatives, and other exotic species.

Based on those baseline genetic variability data, we design a targeted cyt*b* metabarcode assay to discern and identify silver carp, bighead carp, and their cyprinid relatives, in order to facilitate early detection and tracking of species and possible hybrids at all life stages, using Illumina MiSeq high-throughput sequencing (HTS). The assay is designed to also simultaneously distinguish among silver carp haplotype variants, for use in population comparisons. We test our assay‘s application for discerning and identifying taxa and possible introduction vectors using eDNA-containing water sampled from 48 bait shops in the Lake Erie, Lake St. Clair, and Wabash River watersheds. Overall, our investigation aims to provide and apply population genetic, evolutionary phylogenetic analysis, and systematic biological knowledge of genetic variation in silver carp and its cyprinid relatives to interpret and understand invasion dynamics, pathways, and progression.

## Materials and methods

### Sample collection

Tissue samples from silver carp individuals were collected by us, our laboratory members, federal or state agencies, and university collaborators (see Acknowledgments). Collections were made under their state or federal collection permits, using their protocols, and under the University of Toledo’s IACUC protocol #205400, “Genetic studies for fishery management” to CAS. Samples included 11 collection areas (Table 1), representing much of the silver carp’s North American range (Fig 1). Bighead carp also were sampled, along with black, common, and grass carps to provide comparative data from related species. Morphological characters, including gill-raker structure, were used to distinguish silver from bighead carps and discern possible hybrids (see [33]). Samples were labeled, stored in 95% EtOH, and archived at CAS’ G3 lab in the NOAA Pacific Marine Environmental Laboratory.

**Table 1:** Population samples, locations, and numbers of silver carp analyzed per marker.

Population Sample	Location	*N*μ_sats_	*N*_cytb_	*N*_COI_	*N*_concat_	*N*_S7_
**Illinois River**	*Total*	*85*	*62*	*69*	*60*	*2*
IL1	LaGrange, IL	10	12	16	12	0
IL2	Marseilles, IL	50	24	26	24	0
IL3	Chillicothe, IL	25	26	27	24	2
**Wabash River**	*Total*	*54*	*42*	*49*	*39*	*3*
WA1	Lafayette, IN	44	37	39	34	3
WA2	Vincennes, IN	5	1	5	1	0
WA3	New Harmony, IN	5	4	5	4	0
**Missouri River**	Blair, NE	50	29	30	29	3
**Upper Mississippi River**	*Total*	*60*	*33*	*34*	*33*	*4*
UM1	Warsaw, IL (Pool 20)	10	9	10	9	2
UM2	Grafton, IL (Pool 26)	50	24	24	24	2
**Lower Mississippi River**	*Total*	*60*	*38*	*38*	*37*	*7*
LM1	Laketon, KY	10	10	10	10	1
LM2	Vicksburg, MI	50	28	28	27	6
*Total*		*309*	*204*	*220*	*198*	*19*

Numbers of individuals analyzed for microsatellites (μsats) and DNA sequences of: cytochrome *b* (cyt*b*), cytochrome oxidase I (COI), concatenated (concat) mitochondrial DNA (both cyt*b* and COI), and nuclear single-copy ribosomal protein S7 gene intron 1 (S7).

### Population genetic data

Genomic DNA was extracted and purified from the EtOH fixed tissues using DNeasy Blood and Tissue Kits (Qiagen Inc., Valencia, CA USA), quality checked on 1% agarose mini-gels stained with ethidium bromide, and assessed with a Nanodrop spectrophotometer (Thermo Scientific, Bothell, WA, USA). Genetic variation was analyzed at 10 μsat loci, including *Hmo1* and *Hmo11* from Mia *et al*. 2005 [77], *Ar201* from Cheng *et al*. 2007 [78], and *HmoB4*, *B5*, *D8*, *D213*, *D240*, *D243*, and *D246* from King *et al*. 2011 [79] for 309 individuals (Table 1). Representative subsets, which included silver carp from all populations (Table 1) and those differentiated by μsats, were sequenced for the mtDNA COI and cyt*b* genes, along with the S7 gene intron 1 (S7).

For the μsats, 10μL polymerase chain reactions (PCRs) contained 0.35 units AmpliTaq DNA polymerase (ABI; Applied Biosystems™, Foster City, CA, USA), 1X GeneAmp PCR buffer I (ABI), 80μM total dNTPs, 0.4mM spermadine, 0.52μM of each primer, and 2μL of ≥30ng/μl DNA and ddH_2_O. PCRs were conducted on C1000™ thermal cyclers (Bio-Rad Laboratories, Hercules, CA, USA) with 2 min initial denaturation at 94°C, followed by 39 cycles of 40 sec at 94°C, 40 sec annealing (at 52°C for primers of Mia *et al*. (2005) [77], 58°C for Cheng *et al*. (2007) [78], and 56°C for King *et al*. (2011) [79]), and 1 min 72°C extension, capped by 10 min final 72°C extension. Products were diluted 1:50 with ddH_2_O, with 2μL added to 13μL formamide and ABI GeneScan™–500 LIZ size standard solution, loaded into 96-well plates, denatured for 2 min at 95°C, and analyzed on our ABI 3130xl Genetic Analyzer with GeneMapper 4.0 software (ABI). Output profiles were checked manually to confirm allelic size variants.

We amplified and sequenced 992 nts of the mtDNA cyt*b* gene, using the primers Song-F (5'–GTGACTTGAAAAACCACCGTTG–3') [80] and H5 (5'–GAATTYTRGCTTTGG-GAG–3') [81] and 549 nts of the mtDNA COI gene, with COIFF2d (5'–TTCTCCACCAACCACAARGA YATYGG–3') and COIFR1D (5'–CACCTCAGGGTGTC CGAARAAYCARAA–3') [82]. PCRs contained 25μL of 1.25 units AmpliTaq DNA polymerase, 1X GeneAmp PCR Buffer I, 250μM dNTPs, 0.5uM (cyt*b*) or 1μM (COI) of each primer, and 2μL of ≥30ng/μL of DNA template, and ddH_2_O. Conditions were 3 min at 94°C, followed by 34 cycles of 95°C for 30 sec, 40 sec at 50°C, and 72°C for 45 sec, capped by 5 min at 72°C. We also sequenced the S7 intron using S7RPEX1F and S7RPEX2R following Chow and Hazama 1998 [83] for a representative subset of silver carp (*N* = 19), which included randomly selected individuals for all mtDNA haplotypes (Table 1), along with bighead carp, and other carp species (see Results). All sequence data were collected on subsets of the individuals used for μsat analysis.

PCR product aliquots (4μL) were visualized on 1% agarose mini-gels stained with ethidium bromide and successful reactions purified with QIAquick PCR Purification Kits (Qiagen) and quantified via Nanodrop. Sanger DNA sequencing was outsourced to the Cornell University Life Sciences Core Laboratories Center (http://www.biotech.cornell.edu/brc/genomics-facility) and MCLAB (http://www.mclab.com/DNA-Sequencing-Services.html), which used ABI Automated 3730 DNA Analyzers. Sequences were quality scored, manually checked, and aligned by us with CODON CODE ALIGNER v7.01 (CodonCode Corp., Centerville, MA, USA). Additional DNA sequences were mined from N.I.H. GenBank (S1 Table).

### Microsatellite analyses

All loci were evaluated for linkage disequilibrium and conformance to Hardy-Weinberg equilibrium (HWE) expectations, using the Markov Chain Monte Carlo (MCMC) procedure with 10,000 dememorizations, 1,000 batches, and 10,000 iterations per batch in GENEPOP v4.0 [84]. Significance values were adjusted with standard Bonferroni correction [85]. Possible scoring errors, large allele dropout, stuttering, and/or null alleles at each locus were evaluated using MICRO-CHECKER v2.2.3 [86].

Genetic diversity measures, including the number of alleles per locus (*N*_A_), observed heterozygosity (*H*_O_), and allelic richness (*A*_R_; adjusted for sample size using rarefaction) were calculated in *F*STAT v2.9.3.2 [87], their standard errors (±S.E) with EXCEL (Microsoft, Redmond, WA), and the significance values (*p*<*a*) adjusted with standard Bonferroni correction. [85]. Significant differences in *H*_O_ and *A*_R_ were evaluated using paired two-tailed *t*-tests in R v3.2.1 [88]. Number of private alleles (*N*_*PA*_) per locus, i.e., those appearing unique in a population sample, were identified with CONVERT v1.31 [89]. The percentage of private alleles (*P*_*PA*_) was the number of private alleles in a given sample divided by its total number of alleles, using rarefaction representation in ADZEv1.0 to adjust for sample size disparity [90]. COLONY v2.0.6.1 was used to test for the respective presences of full and half siblings in the samples [91]. The possible influence of selection was evaluated using the outlier method of Beaumont and Nichols 1996 [92] in LOSITAN [93].

Pairwise genetic divergences were calculated between all population samples, and separately between the two invasion fronts at the Illinois and Wabash Rivers with the *F*_ST_ analog *θ*_ST_ [94] in *F*STAT, which is regarded as appropriate for analyses of high gene flow species, small sample sizes, and unknown number of subpopulations [95–97], and to facilitate comparisons with other studies. Since *F*-statistic estimates assume a normally distributed data set and may be influenced by sample size [98], we additionally conducted pairwise exact tests of differentiation (χ^2^) in GENEPOP, using MCMC chains of 10,000, 1000 batches, and 10,000 iterations. Probabilities for both types of pairwise genetic divergences were assessed with sequential Bonferroni correction [99].

Microsatellite DNA variation for all individuals that possessed a highly divergent mtDNA haplotype (“H”) was statistically compared against a group that included all of the other haplotypes (see Results and Table 1), to better match the size of the comparative datasets. We also ran the μsat variation of “H” group against the data set containing all other individuals (i.e., the entire data set).

Population relationships from μsat variation were visualized using three-dimensional factorial correspondence analysis (3D-FCA) [100] in GENETIX v4.05 [101]. Bayesian STRUCTURE v2.3.2 [102] analyses evaluated whether, and how many, discrete genetic groups were represented. Ten replicates were conducted for *K* = 1–6 genetic groups, with burn-ins of 50,000 and 100,000 iterations, whose relative support was evaluated with delta *K* [103] in STRUCTURE HARVESTER [104]. Assignment tests were conducted with GENECLASS2 [105] for all populations sampled, and separately for the invasion fronts (Illinois and Wabash rivers), using the “enable probability computation” option, the simulation algorithm from Paetkau *et al*. 2004 [106], and 100,000 simulated individuals.

### DNA sequence analyses

We evaluated mtDNA sequence variation in the cyt*b* and COI regions, both separately and together as concatenated sequences (Table 1), adding available complete gene sequences from NIH GenBank (https://www.ncbi.nlm.nih.gov/genbank/; detailed in S1 Table). Haplotype relationships are depicted with TCS haploptype networks [107] in POPART (http://popart.otago.ac.nz), in reference to four bighead carp haplotypes. ARLEQUIN calculated numbers of haplotypes (*N*_H_), haplotypic diversity (*h*), numbers and proportions of private haplotypes (*N*_PH_ and *P*_PH_) per sample, and pairwise divergences using *θ*_ST_ and exact tests [108]. All of the highly divergent (“H”) haplotype silver carp individuals and representatives of all other haplotypes and populations also were sequenced for the nDNA S7 intron 1 (see Results and Table 1). The purpose was to determine whether their nDNA genome also was genetically divergent.

Relative percentage of mtDNA concantanted mtDNA haplotypes per population was illustrated using a stacked bar graph, drawn with R. Evolutionary relationships among silver carp sequence variants were evaluated with Bayesian phylogenetic trees in MRBAYES v3.2.6 [109], in comparison with bighead carp, grass carp, black carp, and common carp, with the latter as the selected outgroup. These used the GTR + I + Γ model of substitution, a relaxed molecular clock, the rate at which the variance of the effective branch length increased over time, and a default prior exponential distribution rate = 10.0. The MCMC was run for 100,000 generations to calculate support values for branch nodes. Consensus trees were visualized with FIGTREE v1.4.3 (http://tree.bio.ed.ac.uk/software/figtree/).

### Design and testing a diagnostic assay for silver, bighead, and other invasive carps

Using the mtDNA sequence variation discerned for the cyt*b* gene, we developed primers from conserved aligned sequence regions (see Results) that bracketed a diagnostic region of sequence variation, which differentiated among silver carp (and many of its haplotypes), bighead carp, and other invasive carp species for use in a targeted metabarcode HTS assay. The purpose was to facilitate detection, species identification, population variation, and quick, accurate differentiation in field samples.

Our assay was used to test for and evaluate the possible cryptic presence of silver carp (including haplotype variants), bighead carp, and other invasive cyprinid species in retail bait shops, as a pilot study for a larger ongoing project. We evaluated water samples and live bait from 48 bait shops located in the Lake Erie, Lake St. Clair, and Wabash River watersheds in 2016 and 2017. Approximately two dozen bait fishes were purchased from each shop, which were immediately sacrificed in the parking lots using our IACUC protocol. The water containing the fishes was drained into a sterile container, placed on ice, and then frozen at -80°C in the laboratory.

### Conducting the targeted HTS assay

We briefly thawed the water samples on ice, and centrifuged 250 ml from each at 4500 rpm and 4°C, for 45 min. The supernatant was discarded and the pellet, containing extra and intracellular DNA and debris, was resuspended in 95% EtOH and stored at -20°C. DNA was extracted within 24 hr with Qiagen DNeasy kits following the manufacturer’s protocol, except that two AW 1 and 2 buffer washes were performed. The Zymo OneStep PCR Inhibitor Removal Kit (Zymo Genetics, Seattle, WA) was used to alleviate PCR inhibition.

Primers contained spacer inserts to increase the diversity of the HTS libraries [110]. 25μl PCRs comprised 1X Radiant TAQ Reaction Buffer (Alkali Scientific Inc., Ft. Lauderdale, FL), 3mM MgCl2, 1mM total dNTPs, 0.6mM of each primer (with spacer inserts and an Illumina, MiSeq sequencing primer tail), and 1.25 units of Radiant TAQ polymerase. Conditions were 2 min at 95°C, followed by 40 cycles of 95°C for 45 sec, 56°C for 30 sec, and 72°C for 45 sec, capped by 3 min at 72°C. The final PCR used the prior step’s column-cleaned product as its template (2μl) and incorporated Nextera paired end indices (Illumina, kit FC-121-1011), which included P5 and P7 adaptor sequences for binding the prepared library onto the flowcell’s surface. Adding unique combinations of forward and reverse indices permitted multiple samples to be pooled together in each lane. Each reaction contained no template controls (NTCs), of which only those free of NTC amplification were used for libraries (see below). After column clean up, products were sized and quantified by us on a 2100 Bioanalyzer (Agilent Technologies), and concentrations of pooled products were measured with a Qubit fluorometer (Invitrogen). Pooled samples were run with 2X 300 base pair V3 chemistry. An additional 40–50% PhiX DNA spike-in was added to improve data quality of low nt diversity samples [110]. Each HTS run contained positive control mixtures of equal volumes of DNA extractions from 10 marine fish species (not present in the Great Lakes), which had been previously Sanger sequenced, to estimate sequencing error and help validate results [111]. Illumina MiSeq was performed by Ohio State University’s Molecular and Cellular Imaging Center in Wooster, OH, USA (http://mcic.osu.edu/).

### High-throughput sequencing data analyses

Custom bioinformatic scripts in PERL v5.26.1 trimmed primers and removed any sequences having a different spacer insert (which might result from index hopping if a few sequences with a different index became incorporated into an HTS library [112]), or were of the wrong length (i.e., short primer dimer sequences in the FASTQ files of the HTS runs; [113]). Trimmed sequences then were merged, dereplicated (grouped by 100% sequence similarity), and any chimeras eliminated with DADA2 [114], which employed a denoising algorithm with a Poisson model to calculate error probabilities at each nt position. This removed variant nts having a lower percentage representation than the predicted threshold. The resultant screened sequences are termed amplicon sequence variants (ASVs). Unexpected ASVs remaining in the positive controls after merging in DADA2 would be attributable to PCR and/or sequencing error, or sequence-to-sample mis-assignment (via index hopping). The greatest percent representation of any single erroneous ASV was set as the cutoff. If an ASV in a sample occurred at a lower frequency than this cutoff, then it was removed. Remaining ASVs were queried using the Basic Local Alignment Search Tool (BLAST; https://www.ncbi.nlm.nih.gov/) from the command line against a custom database containing cyt*b* sequences of all fishes native to the Great Lakes basin [115] and non-native species that have been documented or have been predicted to possibly be introduced in the future [116]. All BLAST results with the lowest e value (best match) per species were summarized with a custom PERL script that also grouped together multiple detections in a sample. Samples with positive silver carp detections (see Results), were re-sequenced on a separate run using spacer inserts that did not share a forward or reverse index, markedly reducing possibility of undetectable index hops.

### Data accessibility

All μsat allele data, mtDNA haplotypic frequencies, and custom HTS analysis scripts are deposited in the DRYAD public data base (doi:10.5061/dryad.92h1f12). DNA sequences discerned in our study are in GenBank (Accessions: cyt*b*, MH938821–32 and MK205185–86; COI, MH938813–20; and S7, MH938813–43). All HTS data are in the NCBI Sequence Read Archive (BioProject: PRJNA502563. Accessions: SAMN10349832–925). All data generation methods are detailed in protocols.io (dx.doi.org/10.17504/protocols.io.u6zezf6).

## Results

### Microsatellite diversity, divergence, and population structure

Results indicated no linkage disequilibrium, null alleles, or selection. A single locus (*Hmo-B4*) that did not conform to Hardy-Weinberg equilibrium expectations was removed from further analyses. There were no differences in genetic diversity and *θ*_ST_ values when population samples were pooled regionally (e.g., all samples from the Lower Mississippi together, *N* = 60) versus separately for individual sampling locations (Table 2; Fig 1). Thus, our results are presented with regional pooling to best depict overall geographic patterns across the range, encompassing larger sample sizes. Four pairs of full silver carp siblings were identified by COLONY, whose inclusion or exclusion did not significantly alter the *θ*_ST_ values; we presented the complete data here. One individual from three of the sibling pairs was collected in the Illinois River, and both individuals of the fourth pair also occurred there. Of the three located in separate populations, the other siblings respectively were found in the Lower Mississippi River, Upper Mississippi River, and the Missouri River. A total of 509 pairs of half siblings were identified, constituting 0.5% of the possible pairs and representing all possible combinations of population areas. Statistically fewer of the half sibling pairs (*t*-test *p* = 0.008*) co-occurred within the same population (19.8±4.85) versus in different populations (41.0±4.07), indicating high dispersal.

**Table 2:** Genetic diversity of silver carp population samples.

Population	*N* for	* *	*H*_*O*_	*A*_*R*_	* *	* *	*N* for		* *	* *	* *	* *
Sample	μsats	*NA*	±S.E.	±S.E.	*P*_*A*_	*P*_*PA*_	mtDNA	Haps	*H*_*D*_	*N*_*H*_	*P*_*H*_	*P*_*PH*_
**Illinois River**	**85**	**57**	**0.57**	**5.7**	**7**	**0.12**	**60**	**A,B,G**	**0.51**	**3**	**0**	**0**
**(IL)**			**±0.05**	**±0.7**					**±0.03**			
**Wabash River**	**54**	**58**	**0.6**	**6.3**	**3**	**0.05**	**39**	**A,B,H**	**0.54**	**3**	**0**	**0**
**(WA)**			**±0.04**	**±0.7**					**±0.03**			
**Missouri River**	**50**	**53**	**0.58**	**5.9**	**1**	**0.02**	**29**	**A,B,H**	**0.49**	**3**	**0**	**0**
**(MO)**			**±0.06**	**±0.7**					**±0.07**			
**Upper Mississippi**	**60**	**54**	**0.6**	**5.7**	**0**	**0**	**33**	**A,B,F,H**	**0.57**	**4**	**1**	**0.25**
**R. (UM)**			**±0.06**	**±0.7**					**±0.05**			
**Lower Mississippi**	**60**	**54**	**0.6**	**5.9**	**4**	**0.07**	**37**	**A-E,G,H**	**0.64**	**7**	**3**	**0.43**
**R. (LM)**			**±0.06**	**±1.0**					**±0.06**			
**Total**	***309***	***70***	***0*.*6***	***6*.*1***	***15***	***0*.*21***	***198***	**A-H **	***0*.*54***	***8***	***4***	***0*.*5***
** **	*** ***	*** ***	***±0*.*14***	***±0*.*7***	*** ***	*** ***	*** ***	** **	***±0*.*01***	*** ***	*** ***	*** ***

Numbers analyzed per population *(and totals*, *in italics)* for microsatellites (*N* for μsats) or mtDNA (*N* for mtDNA), number of alleles or number of concatenated haplotypes (*N*_A_, *N*_H_), observed heterozygosity or haplotypic diversity (*H*_O,_ *H*_*D*_) ± standard error (S.E.), allelic richness (*A*_R_) ±S.E., number of private alleles or private haplotypes (*P*_A_, *P*_H_), proportion of alleles or haplotypes that are private to the sample (*P*_PA_, *P*_PH_). Haplotypes per population are lettered “A–H”.

A total of 70 μsat alleles were discerned for silver carp (Table 2), with the most occurring at the invasion fronts, including 57 in the Illinois River and 58 in the Wabash River, versus 54 for the Lower Mississippi River population. Observed heterozygosity was 0.60 across all populations, ranging from 0.57 in the Illinois River, to 0.60 in the Wabash River and the Upper and Lower Mississippi rivers; these values did not significantly differ. Allelic richness appeared highest in the Wabash River front population at 6.3 and next highest in the Lower Mississippi River at 5.9, ranging down to 5.7 in the Illinois and Upper Mississippi rivers. No significant differences in allelic richness occurred among the populations. A total of 15 private alleles were identified, constituting 21% of the overall alleles (Table 2). Of these, the Illinois River invasion front possessed the most (seven, totaling 12% of its alleles), followed by the Lower Mississippi River (four at 7%), the Wabash River (three at 5%), and the Missouri River (one at 2%).

Significant genetic divergences distinguished all but just two pairs of populations, assessed by exact tests, indicating overall population structure (Table 3). The two exceptions were similarities between the Upper Mississippi River versus populations in the Missouri and the Illinois rivers. Analyses using *θ*_ST_ divergences revealed four significant differences, between the Lower Mississippi River population versus the Missouri, Illinois, and Wabash rivers, as well as between the Upper Mississippi and Wabash rivers (Table 3A). The two invasion front populations, the Illinois and Wabash rivers, significantly diverged in all analyses (Table 3B).

**Table 3:** Pairwise divergences, based on nuclear DNA microsatellite data, with *θ*_ST_ (below diagonal) and exact tests (above) for: (A) all populations sampled and (B) the invasion front populations alone.

A					
Population Sample	IL	WA	MO	UM	LM
**Illinois R. (IL)**	~	*	*	NS	*
**Wabash R. (WA)**	0.009*	~	*	*	*
**Missouri R. (MO)**	0.005	0.008	~	NS	*
**Upper Mississippi R. (UM)**	0.001	0.010*	0.001	~	*
**Lower Mississippi R. (LM)**	0.007*	0.009*	0.012*	0.006	~
**B**	** **	** **			
**Population Sample**	**IL**	**WA**			
**Illinois R. (IL)**	~	*			
**Wabash R. (WA) **	0.009*	~			

NS (or no asterisk) = not significant

* = significant after sequential Bonferroni correction.

Silver carp that had a highly divergent mtDNA haplotype (designated “H”; see below section, “Mitochondrial and nuclear DNA sequence haplotypes”) did not significantly differ in μsat composition from other silver carp (including analyses against all other individuals and for a subgroup that included representatives of all other haplotypes (see Table 1), with *θ*_ST_ and exact tests.

**Fig 2:**
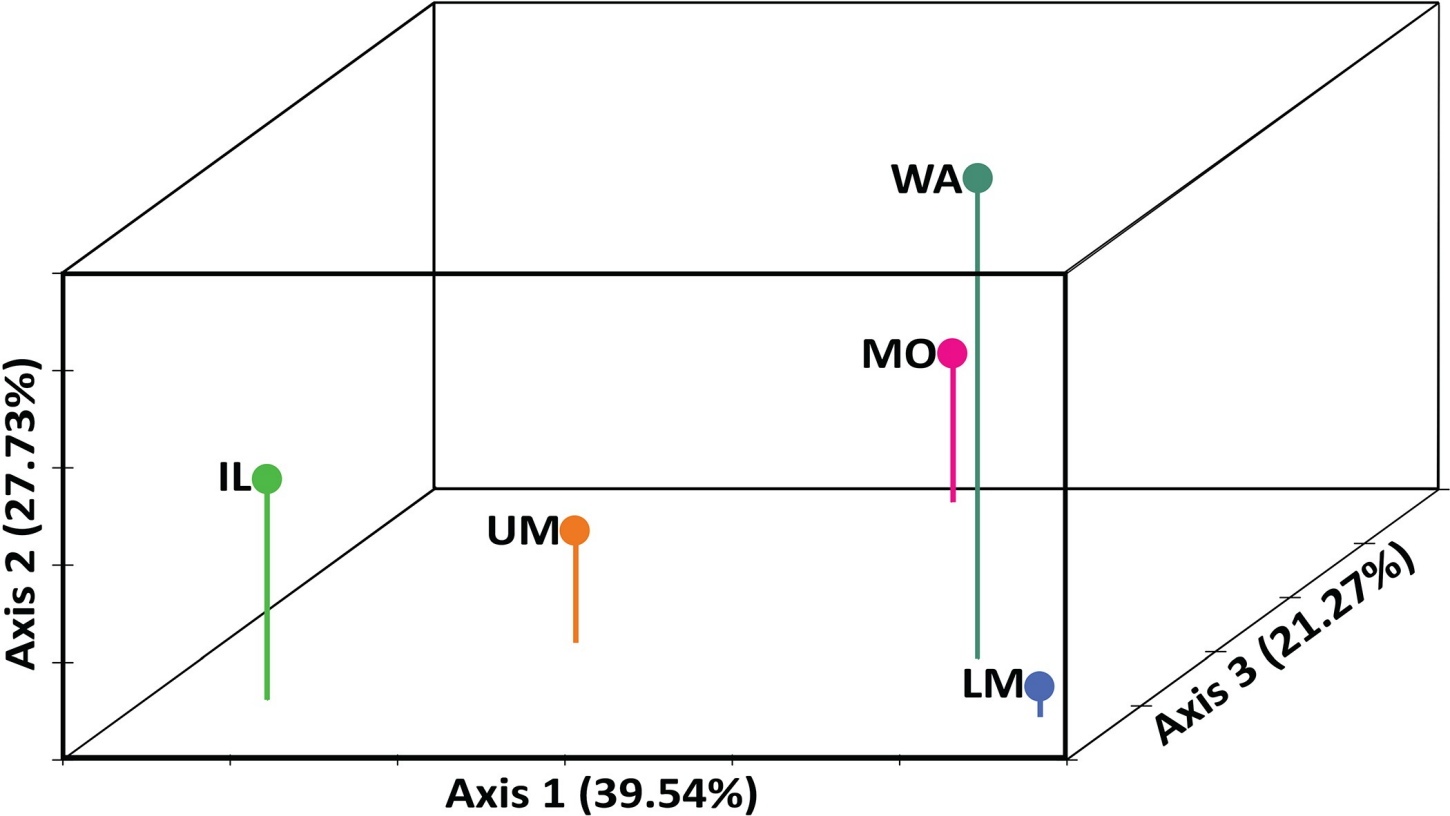
Three–Dimensional Factorial Correspondence analysis illustrating the genetic relationships among silver carp populations, based on microsatellite variation.

The 3-d FCA (Fig 2) explained 88.54% of the data, distinguishing among all populations (Table 3). All appeared widely separated, with the most divergence among the Upper Mississippi, Illinois, and Wabash river samples. STRUCTURE and STRUCTURE HARVESTER best-supported *K* = 2 population groups (not shown), for which distribution plots did not reveal appreciable structure (i.e., all individuals and samples showed approximately equal assignment to both). GENECLASS2 results for the entire data set also discerned overall mixed assignments. However, the GENECLASS2 assignment test between the two invasion front populations, the Illinois versus the Wabash rivers, indicated that 62% and 98% of their individuals respectively self-assigned. Individuals from the Wabash River population thus showed very high self-assignment.

### Mitochondrial and nuclear DNA sequence haplotypes

**Fig 3:**
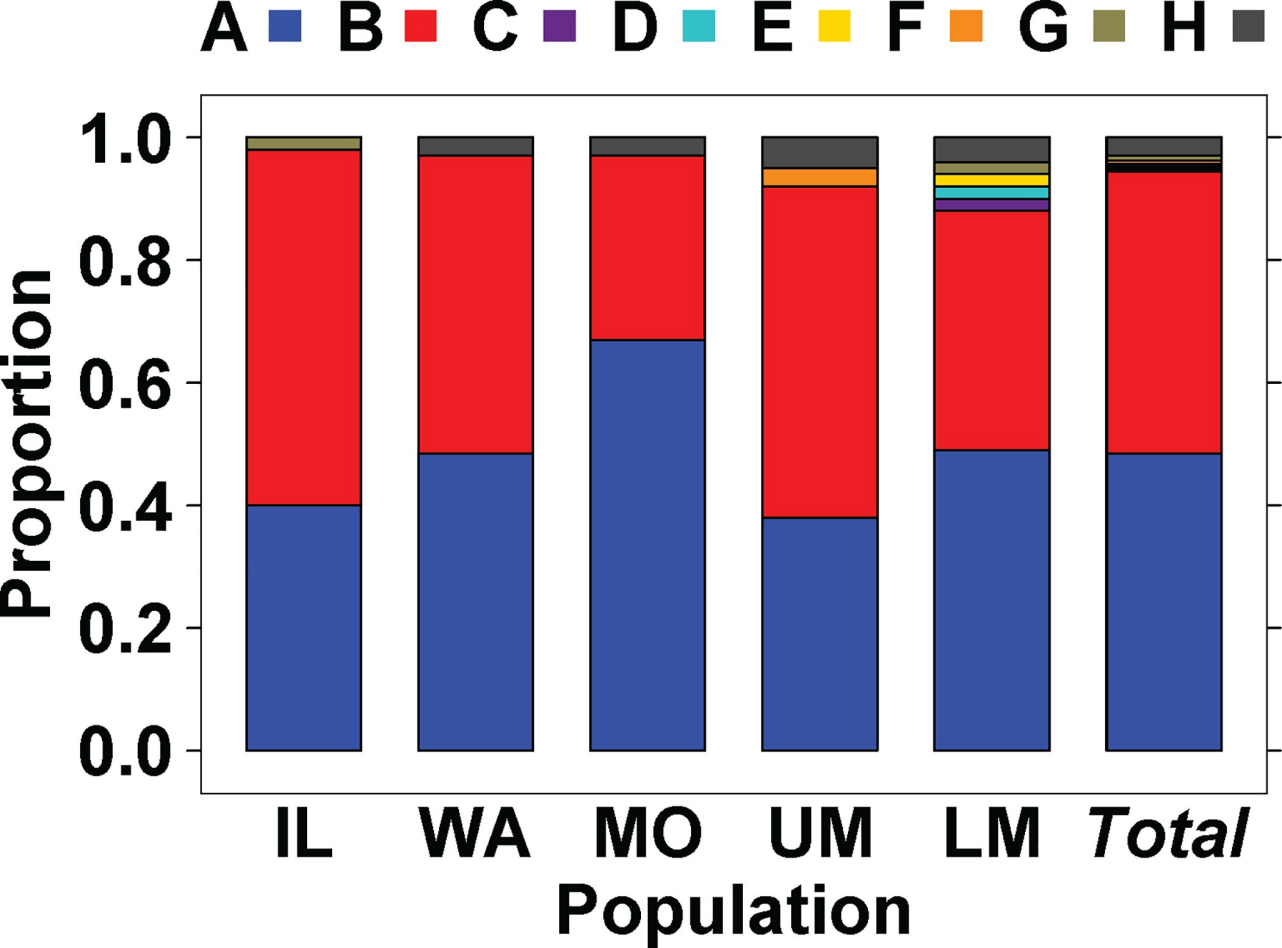
Silver carp mtDNA cytochrome *b* haplotype frequencies in North American population regions. IL = Illinois River, WA = Wabash River, MO = Missouri River, UM = Upper Mississippi River, LM = Lower Mississippi River.

In total, we analyzed 233 mtDNA cyt*b* sequences, along with 248 COI, and 226 concatenated (COI and cyt*b*) sequences for silver carp (Tables 1 and S1). Eight silver carp haplotypes occurred in North America (lettered “A–H” on Fig 3). We analyzed two other cyt*b* haplotypes from GenBank, one from the Yangtze River in China (Accession AF051866, lettered “Q”) and another from the Black River in Russia (AB198974, lettered “R”), which were not available for our sequencing.

**Fig 4:**
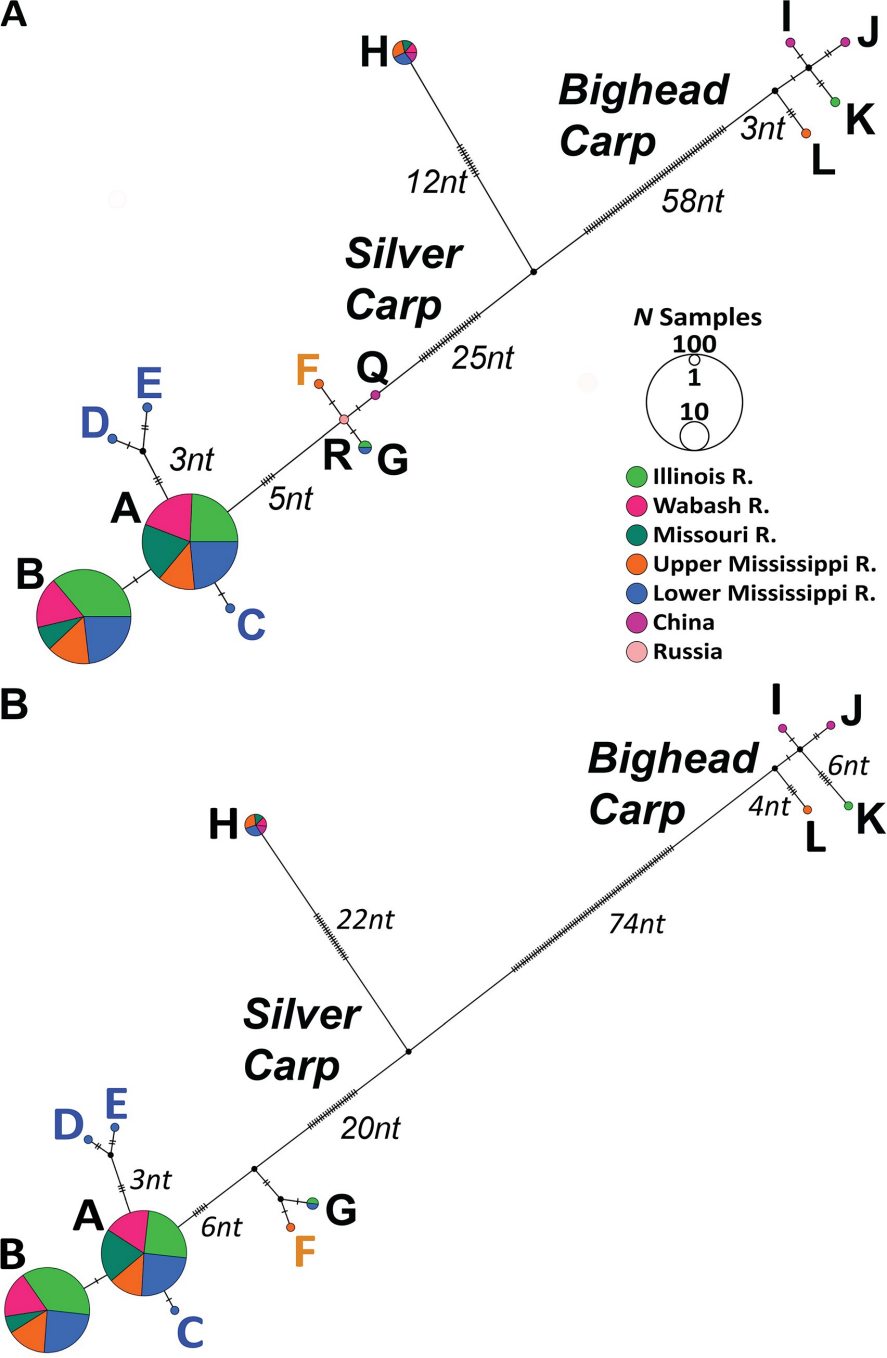
Silver carp TCS mtDNA haplotype networks for (A) cytochrome (cyt) *b* and (B) cyt*b*and COI concatenated mitochondrial DNA sequences. The (A) network for cyt*b* contains GenBank sequences from China and Russia (haplotypes “Q” and “R”, respectively), which are not included in the concatenated network (B) because their COI sequences were unavailable. Bighead carp sequences (“I–L”) are included for comparison. Hash marks denote number of nucleotide substitutions.

**Fig 5:**
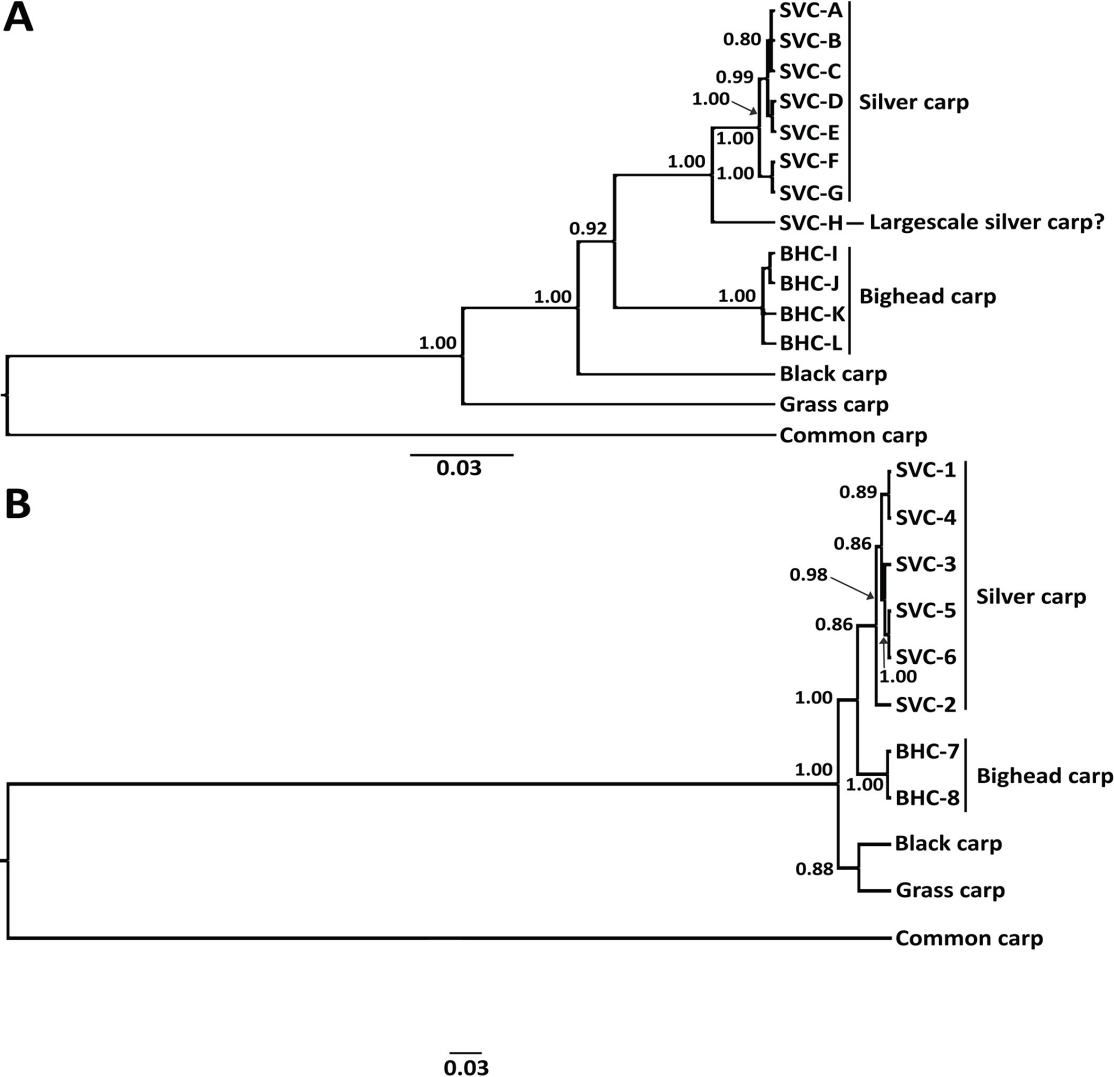
Bayesian phylogenetic trees showing relationships among silver carp (SVC) and bighead carp (BHC) sequence haplotypes, in relation to black, grass, and common carps. (A) Concatenated mitochondrial DNA (mtDNA) tree (cyt*b* and COI genes) with haplotypes lettered. (B) nuclear DNA S7 intron 1 tree, with haplotypes numbered. Incongruence with mtDNA tree implies a historical mtDNA introgression, possibly with the closely related largescale silver carp. SVC S7-“1” contained 12 individuals with haplotypes “A-H” (two of haplotype “A”, one individual each with the “B”, “C”, “D”, “E”, “F”, and “G” haplotypes, and four having haplotype “H”), S7-“2” (one individual with haplotype “A”), S7-“3” (one individual with haplotype “A”), S7-“4” (three individuals with haplotype “B”), S7-“5” (one individual with haplotype “G”), and S7-“6” (one individual with haplotype “H”). Note that one of the six individuals having the mt “H” haplotype did not amplify for S7, and thus was not included. Posterior probability values presented in decimals at nodes.

Genetic relationships among the haplotypes are depicted in Fig 4. The most haplotypes (seven) occurred in the Lower Mississippi River population (which lacked “F” alone) (Fig 3, Tables 2, 4 and 5). Population samples overwhelmingly were dominated by the common haplotypes “A” and “B” (Fig 3), which together comprised >90%, occurred in all populations, and differed by just a single nt (Fig 4). Haplotype “B” was less abundant in the Missouri River than in the other populations. All populations but the Illinois River additionally contained haplotype “H”. Haplotypes “C”, “D”, and “E” were found in the Lower Mississippi River population alone; these were singletons that evolutionary analyses depicted as most closely related to common haplotype “A” (Figs 4 and 5A). Haplotypes “D” and “E” shared a closer relationship with each other. Haplotype “F” (a single individual) was discerned in the Upper Mississippi River population alone (Figs 3 and 4).

**Table 4:** Pairwise divergences using concatenated mtDNA sequences, with *θ*_ST_ (below diagonal) and exact tests (above).

Population Sample	IL	WA	MO	UM	LM
**Illinois R. (IL)**	~	NS	NS	NS	NS
**Wabash R. (WA)**	0.002	~	NS	NS	NS
**Missouri R. (MO)**	0.045*	-0.020	~	NS	NS
**Upper Mississippi R. (UM)**	0.040	-0.010	-0.010	~	NS
**Lower Mississippi R. (LM)**	0.056*	-0.005	-0.022	-0.017	~

NS (or no asterisk) = not significant

* = significant after sequential Bonferroni correction.

**Table 5:** MtDNA concatenated haplotype (Hap) designations and their nucleotide variations for silver carp from the cytochrome *b* and *cytochrome oxidase I* genes.

**Hap**	*117	**141**	**144**	**172**	**183**	**199**	**210**	**216**	**219**	228*	**258**	**309**	**315**	**318**	**348**	**355**	**372**	**420**	**501**	
**A**	A	T	A	G	C	G	T	C	A	A	A	T	A	C	A	T	G	T	C	
**B**	.	.	.	.	.	A	.	.	.	.	.	.	.	.	.	.	.	.	.	
**C**	.	.	.	.	.	.	.	.	.	.	.	.	.	.	.	.	.	.	.	
**D**	.	.	.	.	.	.	.	.	G	.	.	.	.	.	.	.	.	.	.	
**E**	G	.	.	C	.	.	.	.	.	.	.	.	.	.	.	.	.	.	.	
**F**	.	.	.	.	T	.	.	.	.	.	.	.	G	.	.	C	.	.	.	
**G**	.	.	.	.	T	.	.	.	.	.	.	.	.	.	.	C	.	.	.	
**H**	.	C	G	.	.	.	C	T	.	G	G	C	.	T	G	C	A	C	T	
**Hap**	**555**	**564**	**567**	**585**	**598**	**618**	**639**	**669**	**724**	**744**	**745**	**780**	**798**	**807**	**831**	**837**	**840**	**858**	**864**	
**A**	A	C	C	G	C	C	T	C	T	T	C	C	G	A	T	C	T	C	G	
**B**	.	.	.	.	.	.	.	.	.	.	.	.	.	.	.	.	.	.	.	
**C**	.	.	.	.	.	.	.	.	.	.	.	.	.	.	.	.	.	.	A	
**D**	.	.	T	.	.	.	.	.	.	C	.	.	.	.	.	.	.	.	.	
**E**	.	.	T	.	.	.	.	.	.	C	.	.	.	.	.	.	.	.	.	
**F**	.	.	.	.	.	.	.	.	.	.	.	.	A	.	.	T	.	.	.	
**G**	.	.	.	.	.	.	.	.	.	.	A	.	A	.	.	T	.	.	.	
**H**	G	T	.	A	T	T	C	T	C	C	A	T	A	G	C	T	C	T	A	
**Hap**	**901**	**945**	**990**	**1002**	**1005**	**1035**	**1041**	**1050**	**1066**	***147***	***249***	***264***	***354***	***390***	***402***	***492***	***520***	***561***	***579***	***595***
**A**	C	A	A	T	G	T	C	C	G	T	G	A	T	A	C	C	C	T	A	T
**B**	.	.	.	.	.	.	.	.	.	.	.	.	.	.	.	.	.	.	.	.
**C**	.	.	.	.	.	.	.	.	.	.	.	.	.	.	.	.	.	.	.	.
**D**	T	.	.	.	.	.	.	.	.	.	.	G	.	.	.	.	.	.	.	.
**E**	T	.	.	.	.	.	.	.	.	.	.	.	.	.	.	.	.	.	.	.
**F**	.	.	T	.	.	.	.	.	.	.	A	G	.	.	.	T	.	.	.	.
**G**	.	.	T	.	.	.	.	.	.	.	A	G	.	.	.	T	.	.	.	.
**H**	.	G	.	C	A	C	T	T	A	C	A	G	C	C	T	.	T	C	G	C

Nucleotide (nt) positions in the cyt*b* (nt 117–1066) and COI (*147–595; in italics*) genes and substitutions among silver carp haplotypes (lettered “A–H”). Dots denote nts identical to reference haplotype “A”. Haplotype “H” = putative *H*. *harmandi* introgression (all individuals were identical).

* = nt variants discernable by our targeted eDNA assay (117–228). GenBank Accessions: cyt*b*; MH938821–32 and MK205185–86, and COI; MH938813–20.

The invasion front populations of the Illinois and Wabash Rivers contained just three haplotypes each, with the Illinois River containing “G” (as well as a greater abundance of “B”) and the Wabash River with “H” (Figs 3 and 4). The sole differentiations at the population level in mtDNA pairwise divergence tests were between the Illinois River versus the Missouri and the Lower Mississippi rivers, respectively. (Table 4).

Haplotypic diversity across all of our samples was 0.54 and did not significantly differ among populations (Table 2), being highest in the Lower Mississippi River (0.61±0.04), followed by the Upper Mississippi River, and lowest in the Missouri River (0.48±0.07). Most silver carp haplotypes differed by 1–9 nt substitutions from haplotype “A”, with the exception of “H” (whose divergence was much greater; Fig 4). Haplotypes exclusively found outside of North America, i.e., “R” and “Q”, were closest in sequence relationship to our rare “F” and “G” haplotypes (Fig 4).

The haplotype networks revealed a distinctly divergent haplotype “H”, which occurred in all populations except for the Illinois River, and was 47 nt substitutions from the closest silver carp haplotype and 76 from the bighead carp (Fig 4B). The “H” haplotype constituted a basal branch outside of the silver carp clade on the Bayesian phylogenetic tree using concatenated mtDNA sequences, with 1.00 posterior probability support (Fig 5A).

Individuals with the “H” mtDNA haplotype did not vary from other silver carp in their nDNA S7 intron sequences, and lacked a common nDNA genotype or pattern, as shown on the Bayesian phylogenetic tree (Fig 5B). There was no differentiation of those with “H” from the 14 other silver carp individuals examined, which included all other mtDNA haplotypes (“A–G”), i.e., four randomly selected ones with “A”, four with “B”, the singletons “C”, “D”, “E”, and “F”, and both “G” individuals (Table 1). Many of them shared identical sequences for the S7 intron with other “H” individuals and other silver carp haplotypes, while others differed in their S7 sequences alone (S2 Table). For example, 12 individuals possessed the same S7-“1” sequence, including four that had “H”, two with haplotype “A”, and one each with haplotypes “B–G”. Individuals possessing sequences S7-“2” and -“3” each contained one individual of “A”, whereas three individuals with S7-“4” had “B”. A silver carp having the S7-“5” sequence contained “G”, and one with the S7-“6” sequence contained “H” (GenBank Accessions MH938833–43; Fig 5B and S2 Table). Note that one of the “H” individuals did not amplify for S7, and five of the six were analyzed. Thus, the mtDNA “H” variant showed no similar nDNA divergence, in either S7 sequences or μsat allelic frequencies (detailed in above section, “Microsatellite diversity, divergence, and population structure”).

The Bayesian evolutionary trees using mtDNA (Fig 5A) and nDNA sequences (Fig 5B) both showed strong support for the respective silver carp and bighead carp species clades, which each had posterior probabilities of 1.00. There were 21 nt nDNA S7 substitutions separating the silver and bighead carp species (Fig 5B).

### Targeted HTS assay for silver and other carps

The invasive carp assay that we designed, developed, and tested here used the forward primer 5’–TGATGAAAYTTYGGMTCYCTHCTAGG–3’ and reverse primer 5’–AARAAGAATGATGC YCCRTTRGC–3’ to amplify a 135 nt region of the cyt*b* gene that begins at nt114 (Table 5). This region can discern all invasive carp species and many native cyprinids, as well as selected silver carp population variants (S3 Table). Intra-specifically, it differentiates among silver carp haplotypes “B”, “D”, “E”, and the highly divergent “H”, along with “A/C” and “F/G” (with the slash indicating that “A” and “C” group together, as well as “F” with “G”).

PCRs detected eDNA in water from 48 bait shops sampled in the Lake Erie, Lake St. Clair, and Wabash River watersheds in summers 2016 and 2017. We obtained 11,538,299 reads for these libraries (mean per eDNA water sample = 122,109±5,982 reads), of which 7,998,256 (mean = 81,615±3,021) were verifiable; i.e., they contained both primers, the correct spacer (indicating it was not an index hop), and were the correct length (were not attributable to primer dimer; S4A Table). Of those, 6,279,133 (mean = 66,302±2,876) were successfully merged using DADA2 (S4A Table). Following bioinformatic filtering (including removal of ASVs below the cutoff for the positive controls), our assay identified eDNA of several native cyprinids, including emerald shiner *Notropis atherinoides* Rafinesque, 1818, rosyface shiner *N*. *rubellus* (Agassiz, 1850), spottail shiner *N*. *hudsonius* (Clinton, 1824), fathead minnow *Pimephales promelas* Rafinesque, 1820, and golden shiner *Notemigonus crysoleucas* Rafinesque, 1820. Among invasive cyprinids, goldfish eDNA was detected in seven samples, grass carp in four, and common carp in five. Silver carp eDNA occurred in four shops after re-sequencing the libraries that had positive detections. Bighead carp eDNA was found in a single shop (which also contained silver carp eDNA), but whose ASV frequency (0.07% of reads) fell below the cutoff (0.19%). However, its haplotype matched our bighead carp haplotype “L”, which differed from the silver carp “A” and “B” haplotypes, by 14 and 15 nts respectively (S3 Table). This large number of nt differences (>10% of the sequence length) indicated that the eDNA presence of bighead carp was not likely attributable to error.

Of the four bait shop samples that were positive for silver carp, two contained haplotype “B” alone (0.19–0.78% of reads; S4B Table). One store near central Lake Erie possessed both haplotypes “A/C” (2.72% of reads) and “B” (2.02%). A single shop located near the Wabash River, where silver carp are well-established in the wild [72], contained two previously undiscerned “novel” silver carp haplotypes (labeled “N1”, GenBank Accession MK205185, 0.39% of reads and “N2”, MK205186, 0.57% of reads), which each differed from haplotype “B” by single nts (S3 Table). No other silver carp sequences were recovered from that shop. Neither silver nor bighead carps were identified with morphological examination of fish samples purchased from the 48 bait stores. NTCs did not amplify and no detections occurred in the positive controls. Thus, it is highly unlikely that eDNA detection was due to contamination or error.

## Discussion

### Genetic diversity patterns, founder effect, and the “leading edge” hypothesis

Moderate and consistent levels of genetic diversity (measured by heterozygosity and allelic richness) characterize silver carp populations tested here across much of their invasive North American range, based on both μsats and DNA sequences. Genetic diversity at the two invasion front populations did not significantly differ from longer-established core populations, refuting the leading edge hypothesis. Moreover, the two invasive front populations significantly genetically diverged, according to μsat results.

Our findings indicate that silver carp populations possess genetic diversity levels (*A*_R_ = 6.1±0.07, *H*_O_ = 0.60±0.14) that appear comparable to those of other North American invasive fish populations (see [6]). For example, the introduced Eurasian ruffe *Gymnocephalus cernua* (Linnaeus, 1758) in the Great Lakes had much lower allelic richness (*A*_R_ = 3.09±0.91) and similar heterozygosity (*H*_O_ = 0.59±0.03), based on comparable numbers of μsat loci; its lower allelic richness is attributable to a likely single source introduction and founder effect [6]. For neutral loci, such as μsats, allelic richness generally decreases more than does heterozygosity with founder effect and bottlenecks, since rare alleles are more readily affected by drift than are common ones [117, 118]. In comparison, allelic richness for the round goby’s invasion of the Great Lakes (*A*_R_ = 9.01±0.92) was higher and its heterozygosity levels were similar (*H*_O_ = 0.60±0.05) [5] to those determined here for silver carp, again from a similar number of μsat loci.

Population genetic data for silver carp from its native range are scarce, rendering it difficult to directly compare levels of genetic diversity and structure. Farrington *et al*. 2017 [75] described μsat allelic richness of silver carp in North America at 4.7±0.1 versus 6.1±0.2 in its native range, whose values for the former were lower than those discerned here in our results (mean 6.1±0.7). Their mean heterozygosity values in North America appeared slightly greater (0.65) than ours (0.60). These variations likely reflect the respective loci used and the population samples analyzed by them versus by us. Results of both studies indicate that the silver carp showed slight to no evidence of founder effect or bottlenecks in its North American invasion and expansion [75].

Li *et al*. 2011 [76] reported 18 unique mtDNA haplotypes (based on concatenated COI and control region (*D*-loop) sequences) for 94 silver carp individuals from the Illinois and Mississippi rivers, which were undetected in their samples from the native range (*N* = 121; Yangtze, Pearl, and Amur rivers). Their haplotypic diversity was reported to be lower in the Mississippi River basin (0.74) than in the native range (0.95). Since their sequences are not in GenBank or other publicly accessible data bases, we cannot directly compare our data with theirs. Our haplotypic diversity was 0.61 in the Lower Mississippi River, ranging down to 0.48 in the Missouri River, and averaged 0.54 across all of our samples. Our results and those from other studies suggest little to no founder effect for the introduced silver carp in North America.

Some invasive fish populations have been successful in new areas despite reduced genetic diversity levels, in comparison to native ones. For example, the Indo-Pacific lionfishes *Pterois volitans* (Linnaeus, 1758) and *P*. *miles* (Bennett, 1828), which are widely invasive and prolific in the western Atlantic Ocean and the Caribbean Sea, respectively had nine mtDNA control region haplotypes and just a single haplotype in their introduced ranges, versus 36 and 38 in their native ranges [119]. Sacramento pikeminnow *Ptychocheilus grandis* (Ayres, 1854) populations have been highly successful invaders of coastal Californian rivers despite 49.6% reduction in allelic richness due to founding by just four individuals [120]. Independent, allopatric introductions of ruffe into the upper Great Lakes and Bassenthwaite Lake, England showed significant founder effects in μsat allelic richness and heterozygosity levels, with low effective population sizes, and declines in genetic diversity over their 30-year time courses [6]. Chen *et al*. 2012 [121] discerned reduced μsat variation in grass carp introductions on three separate continents in comparison to the native range, with founder effect and bottlenecks were indicated for two of the introductions. The exception was North America, where rapid expansion into the large Mississippi River basin and likely multiple introduction events was hypothesized to have counteracted bottlenecks [121]. Our results suggest that similar rapid expansion may have influenced the genetic diversity of silver carp populations.

Roman and Darling 2007 [12] described that just 37% of introduced populations experienced a significant loss of genetic diversity. For example, most introductions of wakame algae *Undaria pinnatifida* (Harvey, 1873) contained high genetic diversity worldwide, likely stemming from admixture of multiple native strains [122]. Likewise, the ballast water introductions of dreissenid mussels and round goby in the Great Lakes were large and genetically diverse, involving several likely source populations and introduction events, and showed no founder effects [5, 7, 15–16, 18–19]. Such ballast water introductions likely involved up to hundreds or thousands of individual propagules being introduced at a time [12, 123]. These contrast with the more moderate diversity of the silver carp invasion.

The leading edge hypothesis predicts that species with rapidly expanding ranges would possess lower genetic diversity in peripheral populations [20, 124]. This hypothesis has been supported for expansions of several native species from glacial refugia after the Ice Ages [20], including μsat and mtDNA sequence analyses of yellow perch *Perca flavescens* (Mitchill, 1814) populations [125]. The leading edge hypothesis also was supported at three invasive fronts in Ireland of the bank vole *Myodes glareolus* (Schreber, 1780), which showed reduced single nucleotide polymorphisms (SNPs), no accumulation of deleterious alleles, and possible selection for traits involved in immunity and behavior [126]. Lower diversity at the leading edge was implicated in North American expansion areas of the vampire bat *Desmodus rotundus* (É. Geoffroy, 1810) [21].

The leading edge hypothesis has not been supported for some invasions, such as the round goby, whose genetic diversity levels did not differ across the Great Lakes, yet whose populations have remained divergent and respectively stable over their 25 year history [5]. The present study similarly discerns that silver carp does not significantly vary in genetic diversity levels across its invasive range–including at the two fronts tested.

When invasive species spread via jump dispersal, new population areas frequently possess reduced genetic diversity (i.e., founder effect), as was seen with transport of invasive dreissenid mussels from the Great Lakes to smaller lakes to the west (via overland trailered boats) [15, 16] and with the European green crab’s *Carcinus maenas* (Kinnaeus, 1758) spread from the Atlantic to the Pacific coasts of North America [127]. This has not been the case for silver carp here, whose populations appear to have slowly and steadily progressed northward, without “leading” edge drop in genetic diversity. Our results indicate continued maintenance of large population sizes and regular gene flow.

The considerable geographic distances separating silver carp full and half siblings in our study support high dispersal, including within a single generation. Tagging studies of silver carp established in the Wabash River showed that some individuals traveled widely (up to 409 km), while others remained close to the tagging location [128]. This might be an adaptive “strategy” for an interplay between dispersal and site fidelity among various offspring, whose genetic and gene expression components [36] merit future investigation.

Our targeted metabarcoding HTS assay could be applied *en masse* to analyzing eDNA from water or entire plankton samples from net tows (i.e., discerning early life stages of eggs and larvae) to detect new occurrences and dispersal patterns by managers. A targeted assay designed by our laboratory to be specific for the two species of invasive dreissenid mussels, distinguishing between them and many of their haplotypes, has been successfully deployed to simultaneously analyze tens of thousands of veliger larvae to species and population levels from net tows, as well as eDNA water samples [129].

### Genetic divergence patterns and population relationships

Significant population genetic differentiation sometimes occurs across the range of invasive species, which often is attributed to high diversity and multiple founding sources, including the round goby [19], whose population patterns in the Great Lakes have remained stable over 25 years [5]. The ruffe exhibited limited genetic diversity and some population divergence across its 30 year history in the upper Great Lakes, revealing founder effect attributable to a single European source area; it has declined in effective population size and numbers in recent years [6]. In comparison, the zebra mussel’s genetic composition has changed over its three decades in the Hudson River, experiencing several population turn-overs and recolonizations [15, 16], yet has remained consistent in Lake Erie throughout [15, 129]. Some invasions possessed little population genetic structure across their ranges; for example, colonization by Virginia’s warbler *Oreothlypis virginiae* (Baird, 1860) of the Black Hills in South Dakota showed no differentiation, implicating high gene flow and dispersal [130].

In the present investigation, the silver carp displays modest levels of inter-population divergences across its invasive North American range. Population differences are most pronounced between the “core” area of original introduction versus expansion areas. Overall, the silver carp invasion appears to have progressed steadily, without bottlenecks or marked changes in genetic composition. Most alleles are distributed throughout the populations, with some significant differences between the invasion fronts of the Illinois and Wabash Rivers. The latter shows especially high self-assignment, which merits evaluation of genomic expression.

### Genetic history of the invasion, revealed by mtDNA

Interpreting the genetic compositions, diversity levels, and phylogeographic patterns of introduced populations and their sources facilitates understanding how they colonize, expand their ranges (see [118–122, 125–127, 129–131]), and adapt to new environments [2, 36]. The silver carp’s origins are believed to trace to China, based on aquaculture records summarized by Kolar *et al*. 2007 [132] and genetic work by Li *et al*. 2011 [76]. Relation to the latter’s results suggest that origins of the two highest frequency haplotypes found in our study (“A” and “B”), which dominated all of our North American populations, likely trace to the Amur and/or Yangtze Rivers [76]. In our results, “A” was more common in the Wabash River invasion front and “B” at the Illinois River invasion front, accounting for the significant divergence between them.

Sequences of cyt*b* haplotype “Q” from the native Yangtze River in China and “R” from an introduction to the Black River in Russia (both from GenBank) are closest to our rare “F” (Upper Mississippi River) and “G” (Lower Misssissippi River) haplotypes. The singleton haplotypes “C”, “D”, and “E” all occurred in our Lower Mississippi River “core” population. These results suggest that the original population escape of silver carp into the Lower Mississippi River region migrated to found the expansion areas, and that there likely have not been appreciable additional introductions from other overseas sources.

### Introgression with largescale silver carp and bighead carp

In comparison to Farrington *et al*. 2017 [75], who sequenced the entire mitochondrion of 30 silver carp individuals from North America and two from their native range, we sequenced 225 individuals for ~10% of some of the most variable portiion of the mt genome, finding eight haplotypes compared to their six, including a much higher frequency of the highly divergent “H” haplotype (for which they uncovered just a single individual) (Table 5). We included Farringon *et al*.*’s* 2017 [75] sequences in our analyes, except for four of their haplotypes, which either were outside of the cyt*b* and COI gene regions that we sequenced, or had incomplete sequences. With our greater sample size, we discerned that the “H” haplotype is found throughout most of the North American range.

“H” occurred in ~3% of the individuals, which may represent a historic introgression of the silver carp *H*. *molitrix* with the closely related largescale silver carp species, *H*. *harmandi* Sauvage, 1884. Our mtDNA sequence for “H” matches a GenBank haplotype (EU315941) from the Yangtze River. The largescale silver carp *H*. *harmandi* is morphologically distinguishable in possessing larger scales and a deeper body than does *H*. *molitrix* [133]. The largescale silver carp is native to Hainan Island and Vietnam, where silver carp also has been extensively introduced [133]. The two species have widely hybridized in China and Vietnam [110]. Our findings discern that the *H*. *harmandi* haplotype “H” is widespread, occurring in the Lower Mississippi, Upper Mississippi, Missouri, and Wabash rivers, as well as in China.

The S7 sequences and μsat allelic frequencies possessed by the “H” haplotype individuals did not show nDNA differentiation. The “H” haplotype thus appears to have resulted from mtDNA introgression, likely of female *H*. *harmandi* interbreeding with male *H*. *molitrix* in Asia. Introgressed native silver carp likely were present as a small percentage of founders in the North American introduction.

There are 73 nt substitutions separating haplotype “H” from the most similar bighead carp cyt*b* sequence. In comparison, silver carp haplotype “Q”, which is closest to “H”, diverges by 37 nts. Moreover, bighead carp also is very divergent in its S7 sequence.

Studies have reported that wild silver and bighead carp do not widely interbreed in their native range, having developed pre-zygotic reproductive barriers in sympatry [132, 133]. However, the two species often hybridize in aquaculture [77] and hybrids occur across their North American ranges [33, 34]. Lamer *et al*. 2015 [34] employed 57 SNPs and one mtDNA polymorphism to determine that 44.7% of *Hypophthalmichthys* spp. individuals in the Mississippi River Basin have interbred or backcrossed, suggestive of a hybrid swarm. The flow of maternal DNA was found to be biased from silver carp to bighead carp in both the F1’s and bighead carp backcrosses [34]. Greater interspecific recombination in females has been shown by microsatellite genetic linkage mapping of two silver and bighead carp crosses by Guo *et al*. 2013 [134]. Across all of our samples (which we selected to avoid morphological hybrids; see Methods), we identified just a single individual with bighead carp mtDNA, either due to misidentification or hybridization. Thus, it is not likely that our results were influenced by hybridization between bighead and silver carps.

Given our objective to analyze the population genetic relationships of the silver carp in its invasive range, it is relevant to consider whether and how genetic components from bighead and possibly largescale silver carp may influence them in the future. It is possible that hybridization has acted to enhance invasive success. Our targeted HTS assay can be applied to track these mtDNA genotypes *en masse*, while genomic techniques can be used to evaluate the relative fitness of variants.

### Application of our targeted silver carp and cyprinid HTS assay

Our assay to detect and identify silver carp and its variants, as well as other invasive carps, and native cyprinid species, has potential wide application. Similar metabarcoding eDNA assays that utilized degenerate primers have been shown to be highly sensitive, detecting down to tens of copies of a target marker [110, 135]. In a similar but much more limited study of six retailers in the Great Lakes, Mahon et al. 2014 [136] detected white perch *Morone americana* with metabarcoding in one bait shop in Michigan, in which only a single individual was observed in the tank. Previously published methods to detect invasive carps have relied on quantitative (q)PCR (e.g., [137]), cannot differentiate haplotypes, and therefore do not provide any information on population genetic diversity.

We did not physically find silver carp in any of the bait stores surveyed, based on morphological examination of purchased samples. However, we discovered silver carp eDNA in the tanks of four shops, indicating likely presence at low densities. In addition, eDNA of bighead carp was identified from one of those shops (albeit below the cutoff frequency based on positive controls). We also found eDNA of other invasive cyprinids. Our eDNA findings highlight the probability that silver and bighead carps are prevalent in the retail trade in the Great Lakes watershed, where they likely blend in with other “minnows” for sale. Releases of live bait by anglers may comprise an important vector for their introduction, meriting further investigation. Silver carp in the Mississippi River did not display patterns associated with jump dispersal [15, 16]. It is therefore not likely that bait release has played a significant role in its spread in the region to date, but might in its potential spread to the Great Lakes.

The eDNA from the store near the Wabash River contained two silver carp haplotypes that were not recovered in our traditional Sanger sequencing and were not referenced in GenBank (search on November 30, 2018). Although those haplotypes were at low frequency, their possibility of being erroneous would stem from the highly unlikely situation of an undetected index hop co-occurring with two separate sequencing errors that were greater than the probabilities calculated from positive controls. This is unlikely due to use of our custom pipeline that removed index hops and a de-noising algorithm that corrected for sequencing error (see Methods). Both haplotypes have single transitional nt mutations, which are common mtDNA cyt*b* mutations [138]. These haplotypes may have been undetected by traditional sampling and sequencing due to sample size limitations. This shop is located within the invasive range of silver carp. Thus, it is possible that these new eDNA haplotypes are present in the silver carp Wabash River population.

Further refinement of bioinformatic pipelines and use of PCR replicates would enhance the veracity of rare haplotypes discerned with targeted eDNA HTS assays [139, 140]. In fact, another targeted HTS assay discerned several novel zebra mussel haplotypes via mass sequencing tens of thousands of veliger larvae, which is beyond the scope of traditional individual population genetic studies [129]. Additional eDNA and plankton sampling with our assay should be undertaken in order to verify these silver carp haplotypes (and others), and their respective distributions.

Our assay has potential widespread use for screening bait fishes, as well as with water and plankton samples from the field. The latter will allow inexpensive and accurate identification of eggs and larvae to species and population [129], which often lack diagnostic morphological characters and cannot be visually discerned. Moreover, surveying water and plankton samples using this approach will allow their relative species proportions to be evaluated, as well as yield population genetic variability statistics for ecological and biogeographic comparisons (see [129]).

## Conclusions

The present investigation resolves phylogeographic and genetic diversity patterns across the invasive North American range of silver carp, significantly advancing from prior knowledge using larger sample sizes and a combined population genetic approach. We discern consistent genetic diversity levels across the invasion’s expansion, including at the fronts leading to the Great Lakes, which significantly differ in genetic composition. We discover and describe a widespread and highly differentiated ancient mtDNA haplotype, which likely originated from historic introgression with the largescale silver carp in Asia, predating the North American introduction. The introgressed individuals do not differ from silver carp in nDNA μsats or gene sequences, suggesting that female largescale silver carp reproduced with male silver carp.

There is great interest in preventing entry of silver carp into the Great Lakes system using man-made barriers and the use of genetic tools to detect them at likely introduction points, expansions, or via other possible introduction vectors. Our finding of eDNA from silver carp in bait stores in the Lake Erie and Wabash River watersheds points to another likely introduction vector, as these retailers often source from southerly areas where silver carp are well-established.

Our targeted HTS assay is designed to discern and detect multiple divergent haplotypes, including the introgressed haplotype “H”, as well as distinguish and identify related cyprinid species. It can be used on water samples or with plankton tows, which will yield abundant population genetic information, including at early life stages. At egg and larval dispersal stages, new invasions and expansions may be detected early, when it is more feasible to eradicate or control them. The ability to track changes in species composition and population genetic variation, rather than just single species presence or absence, should significantly enhance our understanding of the successes of invasive carps in colonizing new areas. If silver carp spread into the Great Lakes via natural dispersal, populations likely will possess similar genetic diversity, yet may subtly vary in composition, allowing tracking. The fact that the two invasive fronts analyzed here differ in genetic composition, as well as from the longer-established core area of the lower Mississippi River, shows the influence of genetic drift and possible adaptation. Elucidating these genetic patterns will significantly increase ecological understanding of the relative successes and adaptations of invasions, and aid the rapid identification of new populations and range areas.

## Supporting information

S1 Table Additional samples, GenBank Accession numbers, species, population locations, gene regions, and haplotypes (Hap) included in our mtDNA sequence analyses. (DOCX)

S2 Table S7 nuclear ribosomal protein gene, intron 1, partial sequence haplotypes of silver carp. Nucleotide (nt) positions and substitutions, based on alignment to GenBank Accession AY325777 S7-“1” reference sequence, with dots indicating congruence with S7-“1”. Dashes denote sequence deletion (indels). Variants are GenBank Accessions MH938813–43.
(DOCX)

S3 Table MtDNA sequence alignment of the invasive carp HTS assay region. Alignment shows silver carp (SVC) haplotypes “A–H”, and two “novel” haplotypes “N1” and “N2” recovered with the targeted HTS assay (GenBank Accessions: MK205185–6), bighead carp (BHC), and other invasive cyprinid sequences. Nucleotide positions (above sequence) are based on the complete cytochrome *b* gene, with those differing from silver carp haplotype “A” shown and dots denoting homology.
(DOCX)

S4 Table Summary of targeted assay high throughput sequencing run output for targeted invasive carp HTS assay from 48 bait shops in the Lake Erie, Lake St. Clair, and Wabash River watersheds. Raw sequence reads, trimmed reads (had both primers and were the correct length), the number and percent that DADA2 successfully merged for all samples and those having sequences that matched silver carp (% per haplotype, “A/C”, “B”, “N1”, “N2”). Samples are named with the year and sample number.
(DOCX)
