# Supplementary material for: Invasion genetics of the silver carp Hypophthalmichthys molitrix across North America: Differentiation of fronts, introgression, and eDNA metabarcode detection
Source: PLoS One. 2019 Mar 27;14(3):e0203012. doi: 10.1371/journal.pone.0203012 (PMC6436794; doi:10.1371/journal.pone.0203012)
Supplement: S1 Table — (DOCX) [file pone.0203012.s001.docx]

**S1 Table. Additional samples, GenBank Accession numbers, species, population locations, gene regions, and haplotypes (Hap) included in our mtDNA sequence analyses.**

| **Sample** | **GenBank Accession No.** | **Species** | **Location** | **Cyt*b* Hap** | **COI Hap** | **Concat Hap** |
| --- | --- | --- | --- | --- | --- | --- |
| SRSC1 | KJ746957 | *Hypophthalmichthys molitrix* | IL | A | a | A |
| SRSC2 | KJ746954 | *“ “* | “ | B | a | B |
| svcimar140 | KJ746953 | *“ “* | “ | B | a | B |
| imarsc4 | KJ729076 | *“ “* | “ | B | a | B |
| imarsc7 | KJ746960 | *“ “* | “ | A | a | A |
| ARsc17 | KJ671449 | *“ “* | LM | A | a | A |
| ARsc18 | KJ671450 | *“ “* | “ | B | a | B |
| Jumper | KJ74961 | *“ “* | “ | A | a | A |
| G90sc | KJ679503 | *“ “* | “ | B | a | B |
| MYsc13 | KJ729093 | *“ “* | “ | B | a | B |
| MYsc14 | KJ729094 | *“ “* | “ | B | a | B |
| MYsc9 | KJ729092 | *“ “* | “ | A | a | A |
| OHsc05 | KJ746938 | *“ “* | “ | A | a | A |
| OHsc06 | KJ746939 | *“ “* | “ | B | a | B |
| OHsc11 | KJ746940 | *“ “* | “ | A | a | A |
| s1sc3 | KJ746946 | *“ “* | “ | B | a | B |
| s2sc4 | KJ746948 | *“ “* | “ | B | a | B |
| s2sc5 | KJ746949 | *“ “* | “ | A | a | A |
| sc2s3 | KJ746956 | *“ “* | “ | B | a | B |
| LodgeLab1 | KP013119 | *“ “* | MO | A | a | A |
| SCMOO101 | KJ746951 | *“ “* | “ | A | a | A |
| SCMOO107 | KJ746952 | *“ “* | “ | B | a | B |
| SCMOO97 | KJ746950 | *“ “* | “ | A | a | A |
| PL26sc58 | KJ746943 | *“ “* | UM | A | a | A |
| PL26SC96 | KJ746944 | *“ “* | “ | A | a | A |
| pl26sc97 | KJ746945 | *“ “* | “ | B | a | B |
| SCPool20 | KJ746955 | *“ “* | “ | A | a | A |
| Hm_BlackR2 | AB198974 | *“ “* | Russia | R | NA | NA |
| Hm_Yangtze1 | AF051866 | *“ “* | China | Q | NA | NA |
| Hm_Yangtze2 | EU315941 | *H. molitrix** | China | H | h | H |
| Hm_Yangtze | JQ231114 | *H. nobilis*** | China | I | i | I |
| Hn_Yangtze | EU343733 | *“ “* | “ | J | h | J |
| Hn-Pool20 | KJ746958 | *“ “* | UM | L | l | L |
| Hn-ILAG-441 | MH938829 | *“ “* | IL | K | k | K |
| Black carp | MH938831 | *Mylopharyngodon piceus* | MO |  |  |  |
| Common carp | MH938832 | *Cyprinus carpio* | OH |  |  |  |
| Grass carp | MH938830 | *Ctenopharyngodon idella* | LM |  |  |  |

*GenBank listed as *H. moltrix,* but appears to be *H. harmandi*.

**GenBank listed as *H. moltrix* but has mtDNA of *H. nobilis*.

NA=not available.
