# Supplementary material for: Invasion genetics of the silver carp Hypophthalmichthys molitrix across North America: Differentiation of fronts, introgression, and eDNA metabarcode detection
Source: PLoS One. 2019 Mar 27;14(3):e0203012. doi: 10.1371/journal.pone.0203012 (PMC6436794; doi:10.1371/journal.pone.0203012)
Supplement: S2 Table — Nucleotide (nt) positions and substitutions, based on alignment to GenBank Accession AY325777 S7-“1” reference sequence, with dots indicating congruence with S7-“1”. Dashes denote sequence deletion (indels). Variants are GenBank Accessions MH938813–43. (DOCX) [file pone.0203012.s002.docx]

**S2 Table. S7 nuclear ribosomal protein gene, intron 1, partial sequence haplotypes of silver carp.**

| **Hap** | 150 | 202 | 253 | 273 | 403 | 445 | 462 | 468 | 558 | 577 | 646 | 647 | 648 | 649 | 650 | 658 | 661 | 669 | 732 | 733 | 735 |
| --- | --- | --- | --- | --- | --- | --- | --- | --- | --- | --- | --- | --- | --- | --- | --- | --- | --- | --- | --- | --- | --- |
| **S7-1** | T | C | A | C | G | T | C | G | T | A | - | - | - | C | C | T | C | T | - | - | C |
| **S7-2** | . | T | . | . | . | C | . | A | . | . | - | - | - | - | - | . | . | . | A | C | G |
| **S7-3** | . | . | . | S | R | . | M | . | . | . | - | - | C | C | C | C | . | . | W | C | S |
| **S7-4** | . | . | . | . | . | . | . | . | . | . | - | - | C | C | C | . | . | . | - | - | . |
| **S7-5** | A | T | G | . | A | . | A | . | C | G | - | - | - | C | C | . | T | C | - | - | . |
| **S7-6** | . | . | G | G | A | . | A | . | C | G | C | C | C | C | C | . | T | C | - | - | . |

Nucleotide (nt) positions and substitutions, based on alignment to GenBank Accession AY325777 S7-“1” reference sequence, with dots indicating congruence with S7-“1”. Dashes denote sequence deletion (indels). Variants are GenBank Accessions MH938813–43.
