# Supplementary material for: Invasion genetics of the silver carp Hypophthalmichthys molitrix across North America: Differentiation of fronts, introgression, and eDNA metabarcode detection
Source: PLoS One. 2019 Mar 27;14(3):e0203012. doi: 10.1371/journal.pone.0203012 (PMC6436794; doi:10.1371/journal.pone.0203012)
Supplement: S3 Table — Alignment shows silver carp (SVC) haplotypes “A–H”, and two “novel” haplotypes “N1” and “N2” recovered with the targeted HTS assay (GenBank Accessions: MK205185–6), bighead carp (BHC), and other invasive cyprinid sequences. Nucleotide positions (above sequence) are based on the complete cytochrome b gene, with those differing from silver carp haplotype “A” shown and dots denoting homology. (DOCX) [file pone.0203012.s003.docx]

**S3 Table. MtDNA sequence alignment of the invasive carp HTS assay region.**

| **Sp./Hap** | **115** | **117** | **120** | **126** | **129** | **135** | **136** | **141** | **144** | **147** | **150** | **151** | **153** | **156** | **159** | **162** | **165** |
| --- | --- | --- | --- | --- | --- | --- | --- | --- | --- | --- | --- | --- | --- | --- | --- | --- | --- |
| **SVC-A** | T | A | C | T | C | C | C | T | A | A | C | C | A | C | A | C | T |
| **SVC-B** | . | . | . | . | . | . | . | . | . | . | . | . | . | . | . | . | . |
| **SVC-C** | . | . | . | . | . | . | . | . | . | . | . | . | . | . | . | . | . |
| **SVC-D** | . | . | . | . | . | . | . | . | . | . | . | . | . | . | . | . | . |
| **SVC-E** | . | G | . | . | . | . | . | . | . | . | . | . | . | . | . | . | . |
| **SVC-F** | . | . | . | . | . | . | . | . | . | . | . | . | . | . | . | . | . |
| **SVC-G** | . | . | . | . | . | . | . | . | . | . | . | . | . | . | . | . | . |
| **SVC-H** | . | . | . | . | . | . | . | C | G | . | . | . | . | . | . | . | . |
| **SVC-N1** | . | . | . | . | . | . | . | . | . | . | . | . | . | . | . | . | . |
| **SVC-N2** | . | . | . | . | . | . | . | . | . | . | . | . | . | . | . | . | . |
| **BHC-I** | . | . | T | . | . | . | . | C | G | G | . | . | . | . | . | . | . |
| **BHC-J** | . | . | T | . | . | . | . | C | G | G | . | . | . | . | . | . | . |
| **BHC-K** | . | . | T | C | . | . | . | C | G | . | . | . | . | . | . | . | C |
| **BHC-L** | . | . | T | . | . | . | . | C | G | G | . | . | . | . | . | . | . |
| **Black carp** | . | . | . | . | . | . | . | C | . | . | . | . | G | . | . | . | C |
| **Common carp** | C | . | . | . | . | T | T | C | C | . | . | . | . | . | . | . | C |
| **Goldfish** | . | . | . | . | T | T | . | C | C | . | T | . | . | T | . | T | C |
| **Grass carp** | . | . | . | . | T | . | . | C | . | G | . | T | . | . | G | T | C |
|  |  |  |  |  |  |  |  |  |  |  |  |  |  |  |  |  |  |
| **Sp./Hap** | **168** | **171** | **172** | **174** | **177** | **181** | **183** | **188** | **189** | **195** | **198** | **199** | **200** | **201** | **207** | **208** | **210** |
| **SVC-A** | C | T | G | T | C | A | C | T | T | T | A | G | T | T | C | T | T |
| **SVC-B** | . | . | . | . | . | . | . | . | . | . | . | A | . | . | . | . | . |
| **SVC-C** | . | . | . | . | . | . | . | . | . | . | . | . | . | . | . | . | . |
| **SVC-D** | . | . | . | . | . | . | . | . | . | . | . | . | . | . | . | . | . |
| **SVC-E** | . | . | C | . | . | . | . | . | . | . | . | . | . | . | . | . | . |
| **SVC-F** | . | . | . | . | . | . | T | . | . | . | . | . | . | . | . | . | . |
| **SVC-G** | . | . | . | . | . | . | T | . | . | . | . | . | . | . | . | . | . |
| **SVC-H** | . | . | . | . | . | . | . | . | . | . | . | . | . | . | . | . | C |
| **SVC-N1** | . | . | . | . | . | . | . | . | . | . | . | A | . | . | . | . | . |
| **SVC-N2** | . | . | . | . | . | . | . | C | . | . | . | A | . | . | . | . | . |
| **BHC-I** | . | . | . | . | . | . | . | . | . | A | . | . | . | C | . | C | C |
| **BHC-J** | . | . | . | . | . | . | . | . | . | A | . | . | . | C | . | . | C |
| **BHC-K** | . | . | . | . | . | . | . | . | . | A | . | . | . | C | . | . | C |
| **BHC-L** | . | . | . | . | . | G | . | . | . | A | . | . | . | C | . | . | C |
| **Black carp** | . | . | . | . | T | . | . | . | . | A | . | . | . | C | . | . | C |
| **Common carp** | . | A | . | C | T | . | . | . | C | . | T | A | C | C | . | . | C |
| **Goldfish** | . | A | . | C | T | . | . | . | C | C | T | A | C | C | . | . | C |
| **Grass carp** | T | . | . | C | . | . | . | . | C | A | . | . | . | C | T | . | C |

**S3 Table (continued).**

| **Sp./Hap** | **213** | **215** | **216** | **219** | **222** | **225** | **228** | **231** | **234** | **237** | **240** | **243** | **244** | **246** | **249** |
| --- | --- | --- | --- | --- | --- | --- | --- | --- | --- | --- | --- | --- | --- | --- | --- |
| **SVC-A** | A | A | C | A | T | T | A | A | C | C | C | C | C | A | C |
| **SVC-B** | . | . | . | . | . | . | . | . | . | . | . | . | . | . | . |
| **SVC-C** | . | . | . | . | . | . | . | . | . | . | . | . | . | . | . |
| **SVC-D** | . | . | . | G | . | . | . | . | . | . | . | . | . | . | . |
| **SVC-E** | . | . | . | . | . | . | . | . | . | . | . | . | . | . | . |
| **SVC-F** | . | . | . | . | . | . | . | . | . | . | . | . | . | . | . |
| **SVC-G** | . | . | . | . | . | . | . | . | . | . | . | . | . | . | . |
| **SVC-H** | . | . | T | . | . | . | G | . | . | . | . | . | . | . | . |
| **SVC-N1** | . | G | . | . | . | . | . | . | . | . | . | . | . | . | . |
| **SVC-N2** | . | . | . | . | . | . | . | . | . | . | . | . | . | . | . |
| **BHC-I** | . | . | T | . | C | . | C | G | T | T | . | . | . | . | . |
| **BHC-J** | . | . | T | . | C | . | C | G | T | T | . | . | . | . | . |
| **BHC-K** | . | . | T | . | C | . | C | G | T | T | . | . | . | . | . |
| **BHC-L** | . | . | T | . | C | . | C | G | T | T | . | . | . | . | . |
| **Black carp** | . | . | . | . | . | C | C | . | . | . | . | . | A | C | T |
| **Common carp** | . | . | . | . | . | C | C | . | A | . | T | T | G | . | . |
| **Goldfish** | . | . | T | . | C | C | C | . | A | T | T | T | A | T | . |
| **Grass carp** | G | . | . | C | . | C | C | . | T | . | . | . | . | . | . |

Alignment shows silver carp (SVC) haplotypes “A–H”, and two “novel” haplotypes “N1” and “N2” recovered with the targeted HTS assay (GenBank Accessions: MK205185–6), bighead carp (BHC), and other invasive cyprinid sequences. Nucleotide positions (above sequence) are based on the complete cytochrome *b* gene, with those differing from silver carp haplotype “A” shown and dots denoting homology.
