# Supplementary material for: Invasion genetics of the silver carp Hypophthalmichthys molitrix across North America: Differentiation of fronts, introgression, and eDNA metabarcode detection
Source: PLoS One. 2019 Mar 27;14(3):e0203012. doi: 10.1371/journal.pone.0203012 (PMC6436794; doi:10.1371/journal.pone.0203012)
Supplement: S4 Table — Raw sequence reads, trimmed reads (had both primers and were the correct length), the number and percent that DADA2 successfully merged for all samples and those having sequences that matched silver carp (% per haplotype, “A/C”, “B”, “N1”, “N2”). Samples are named with the year and sample number. (DOCX) [file pone.0203012.s004.docx]

**S4 Table. Summary of targeted assay high throughput sequencing run output for targeted invasive carp HTS assay from 48 bait shops in the Lake Erie, Lake St. Clair, and Wabash River watersheds.**

| **A** |  |  |  |  |  |
| --- | --- | --- | --- | --- | --- |
|  | ***N* reads** | | | |  |
| **All Samples** | **Raw** | **Trimmed** | **Merged DADA2** | **Prop Merged** |  |
| Total | 11,538,299 | 7,998,256 | 6,279,133 | ***--*** |  |
| Mean | 122,109±5,982 | 81,615±3,021 | 66,302±2,876 | 0.83±0.01 |  |
| **B** |  |  |  |  |  |
|  | ***N* reads** | | | | |
| **Sample Code** | **Raw** | **Trimmed** | **Merged DADA2** | **Prop Merged** | **% Silver Carp Haplotype** |
| 2016:14 | 82,334 | 65,460 | 40,726 | 0.62 | 0.78 “B” |
| 2016:30 | 41,276 | 35,111 | 28,715 | 0.82 | 0.33 “B” |
| 2016:41 | 198,302 | 99,510 | 81,399 | 0.82 | 2.71 “A/C”, 2.02 “B” |
| 2017:03 | 111,237 | 52,798 | 47,432 | 0.90 | 0.39 “N1”, 0.57 “N2” |

Raw sequence reads, trimmed reads (had both primers and were the correct length), the number and percent that DADA2 successfully merged for all samples and those having sequences that matched silver carp (% per haplotype, “A/C”, “B”, “N1”, “N2”). Samples are named with the year and sample number.
